# Synthesis and Characterization of Some New *C_2_* Symmetric Chiral Bisamide Ligands Derived from Chiral Feist’s Acid

**DOI:** 10.3390/molecules17055550

**Published:** 2012-05-09

**Authors:** Abdullah M. A. Al Majid, Mohammad Shahidul Islam, Zeid Abdullah Al-Othman, Ahlam F. Al-Salhoob, Assem Barakat

**Affiliations:** Department of Chemistry, Faculty of Science, King Saud University, P.O. Box 2455, Riyadh 11451, Saudi Arabia; Email: zaothman@ksu.edu.sa (Z.A.A.-O.); rhofy29@hotmail.com (A.F.A.-S.); ambarakat@ksu.edu.sa (A.B.)

**Keywords:** resolution of Feist’s acid, bisamide ligand, *C_2_* symmetric

## Abstract

The hemilabile chiral *C_2_* symmetrical bidentate substituted amide ligands (1*R*,2*R*)-**5_a-d_** and (1*S*,2*S*)-**6_a-d_** were synthesized in quantitative yield from (1*R*,2*R*)-(+)-3-methylenecyclo-propane-1,2-dicarboxylic acid (1*R*,2*R*)-**3** and (1*S*,2*S*)-(-)-3-methylene-cyclopropane-1,2-dicarboxylic acid (1*S*,2*S*)-**3**, in two steps, respectively. The chiral Feist’s acids (1*R*,2*R*)-**3** and (1*S*,2*S*)-**3** were obtained in good isomeric purity by resolution of *trans*-(±)-3-methylene-cyclopropane-1,2-dicarboxylic acid from an 8:2 mixture of *tert*-butanol and water, using (*R*)-(+)-*α*-methylbenzyl amine as a chiral reagent. This process is reproducible on a large scale. All these new synthesized chiral ligands were characterized by ^1^H-NMR, ^13^C-NMR, IR, and mass spectrometry, as well as elemental analysis and their specific rotations were measured. These new classes of *C_2_* symmetric chiral bisamide ligands could be of special interest in asymmetric transformations.

## 1. Introduction

Over the past few decades, *C_2_* symmetric chiral amides and sulfonamides have proven to be efficient ligands for several asymmetric transformations [1,2,3,4], due to their great potential of binding with the metal alkoxides, especially with Ti(IV) alkoxides, through the nitrogen atom. Therefore considerable efforts have been devoted to the synthesis of a variety of substituted *C_2_* symmetric chiral amide ligands [5,6,7,8]. Recently a series of new chiral sulfonamides with a rigid cyclohexyl backbone were introduced by Wals and co-workers for the asymmetric addition of diethyl zinc to aldehydes [8]. In addition, all these *C_2_* symmetric chiral amides and sulfonamides are capable of forming five- and six-membered rings with metal chelates, in which the transition states only allow the approach of incoming groups from the less hindered side by blocking the highly hindered face. Moreover, asymmetric addition of organozinc to aldehydes is probably the most successful and still vigorously pursued area in asymmetric C-C bond formation [9,10,11,12,13]. Despite of the enormous success of chiral ligands in asymmetric reactions, a limited number of amides with 1,1'-biaryl backbones are reported for the organozinc addition [14,15,16,17,18,19,20,21,22]. In addition, the rational design of new chiral ligands for enantioselective conjugate alkylation has achieved limited success, presumably due to several factors that have to be taken into consideration. In case of *C_2_* symmetric chiral amide ligands, these factors could be explained as follows: firstly, due to the presence of *C_2_* symmetric axis in the chiral ligand, a number of possible transition states, in particular chiral transformations could be minimized [23]. Secondly, in the amide functional group, the carbonyl group has a potential donor site -NH group and therefore elucidation of their protonation behavior and related phenomena such as hydrogen bonding and Lewis acid complexation has drawn a good deal of attention. An amide molecule may have a dual role of both proton acceptor and donor, conferring a dual nature to the amide functionality [24]. Nevertheless, the size of the chelate ring has also proven to be important, since it controls the orientation of the substituents around the metal center. Hence, the bulkiness of the substituent in the amide ligands could be adjusted by changing the amide chain in order to achieve a better ligand structure for a particular reaction simply by selecting the appropriate stereochemistry and bulkiness [23].

In addition, Ikeda reported that the presence of stereogenic centers on the backbone of the ligands, introduces an extra element of complexity in the ligand structure, and special effect will arise when such ligands are employed in asymmetric catalysis [25]. Therefore *C_2_* symmetric chiral ligands have been used in the past few decades for catalytic asymmetric processes with a high degree of enantioselectivity [26,27,28,29], although chiral amides still remain an attractive choice for highly selective catalytic reactions due to their ready availability and simple reaction conditions. Hence, the development and application of *C_2_* symmetric chiral amides are still interesting and limited. 

In this article, we report the preparation of (±)-Feist’s acid as a chiral precursor in order to introduce a cyclopropane framework into the *C_2_* symmetric chiral ligands. For this purpose we need an effective resolution of this (±)-Feist’s acid. There are many protocols in the literature for the resolution of (±)-Feist’s acid. In the earliest, Doering and Roth described the resolution of Feist’s acid, using *L*-(−)-quinine as the resolving reagent [30], whereas Al-Majid *et al*., used *L*-(−)-menthol as a resolving reagent [31]. Recently, Godfrey *et al*. have used (*R*)-(+)-*α-*methylbenzyl amine as a chiral reagent [32]. Although a generally applicable method is still lacking, our attention, in this context, has focused on the effective modification of (*±*)-Feist’s acid resolution, and the general method for the synthesis of novel *C_2_* symmetric chiral bisamide ligands with a rigid cyclopropane framework.

## 2. Results and Discussion

**Figure 1:**
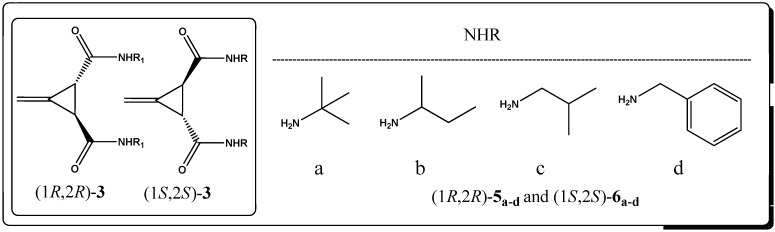
Substituted bis-amide ligands derived from Feist’s acids.

**Scheme 1:**
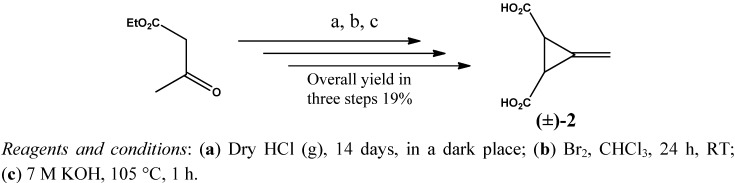
Synthesis of *trans-(±)-*3-methylenecyclopropane-1,2-dicarboxylic acid (±)-**2**.

A series of new chiral bisamide ligands *(1R*,*2R)-***5_a–d_** and (1*S*,2*S*)-**6_a–d_** (Figure 1) has been synthesized from the highly enantiopure Feist’s acids * (1R*,*2R)-***3** and (1*S*,2*S*)-**3**, which were prepared from *trans*-(±)-3-methylenecyclopropane-1,2-dicarboxylic acid (**2**) in very good yield (~92%) using (*R*)-(+)-*α*-methylbenzyl amine as a chiral reagent. Since an optical active Feist’s acid is a commercially available but highly expensive material, therefore it was prepared in house with an overall yield of 19% over three steps, starting from a very cheap and readily available material; ethyl acetoacetate, as described by Goss, Ingold and Thorpe [33], as shown in Scheme 1. 

**Scheme 2:**
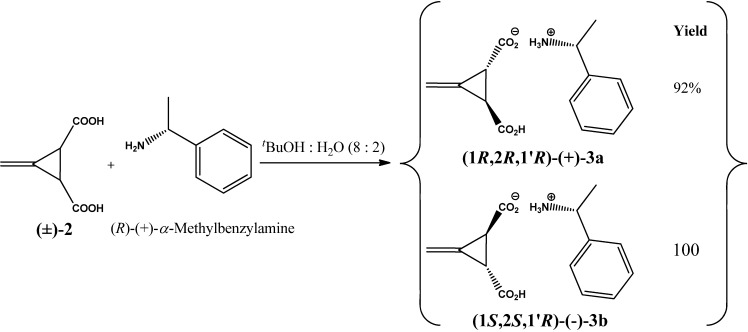
Synthesis of (*R*)-(+)-*α*-methylbenzammonium salt of *trans*-(±)-3-methylenecyclopropane-1,2-dicarboxylic acids{(1*R*,2*R*,1'*R*)-(+)-**3a** and (1*S*,2*S*,1'*R*)-(+)-**3b**}.

In order to obtain enantiopure Feist’s acids *(1R*,*2R)*-**3** and (*1S*,*2S*)-**3** as a precursor, a resolution has been carried out by the reaction between *trans-*(±)-3-methylenecyclopropane-1,2-dicarboxylic acid (±)-**2** and one molar equivalent of (*R*)-(+)-*α*-methylbenzyl amine in aqueous *tert*-butanol (8:2) to give a mixture of diastereomeric ammonium salts of *trans-(±)*-3-methylenecyclopropane-1,2-dicarboxylic acid, as presented in Scheme 2.

On standing overnight at ambient temperature a solid precipitate of (*R*)-(+)-*α*-methyl-benzammonium salt of (1*R*,2*R*)-(+)-3-methylenecyclopropane-1,2-dicarboxylic acid {(1*R*,2*R*,1'*R*)-(+)-**3a**} was obtained in 97.2% yield (1:1) and the (*R*)-(+)-*α*-methylbenzammonium salt of (*1S*,*2S*)-(−)-3-methylenecyclopropane-1,2-dicarboxylic acid (1:1) (1*S*,2*S*,1'*R*)-(+)-**3b**(≈100%) was collected as mother liquor. The yields of isolated compounds (1*R*,2*R*,1'*R*)-(+)-**3a** and (1*S*,2*S*,1'*R*)-(+)-**3b** were measured based on one of the diastereomeric Feist’s acid salts. The melting point and specific rotation of compound (1*R*,2*R*,1'*R*)-(+)-**3a** were found to be 220–222 °C and [*α*]_observed_ + 0.55°, [*α*]^20^_D_ + 110°, (*c*, 0.5%, H_2_O), [lit. [32], [*α*]^23.5^_D_ + 86.1°; (*c*, 0.57%, H_2_O)] accordingly. Further recrystalization of compound (1*R*,2*R*,1'*R*)-(+)-**3a** from *tert*-butanol and water (8:2) was carried out to obtain pure crystals of the (*R*)-(+)-*α*-methylbenzammonium salt of (+)-3-methylenecyclopropane-(1*R*,2*R*)-1,2-dicarboxylic acid (1*R*,2*R*,1'*R*)-(+)-**3a** in approximately 92% yield (based on one of the diastereomeric Feist’s acid salts), m.p. 219–222 °C, [*α*]_observed_ + 0.60°, [*α*]^20^_D_ + 120°, (*c*, 0.50%, H_2_O), [lit. [32], [*α*]^25^_D_ + 94.7° (c, 0.61%, H_2_O)]. The melting point and specific rotation values of compound (1*R*,2*R*,1'*R*)-(+)-**3a** were in accordance with those reported in the literature [32]. 

**Scheme 3:**
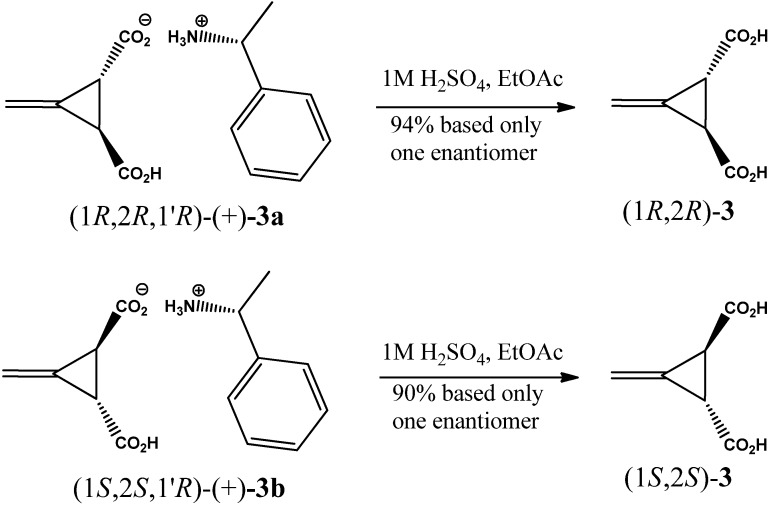
Hydrolysis of (*R*)-(+)-*α*-methylbenzammonium salt of *trans*-(*±*)-3-methylenecyclopropane-1,2-dicarboxylic acids {(1*R*,2*R*,1'*R*)-(+)-**3a** and (1*S*,2*S*,1'*R*)-(+)-**3b**}.

The hydrolysis of the salt (1*R*,2*R*,1'*R*)-(+)-**3a** by using 1 N H_2_SO_4_ in ethyl acetate yielded (1*R*,2*R*)-(+)-3-methylenecyclopropane-1,2-dicarboxylic acid {(1*R*,2*R*)-**3**}, as an off white solid in 94% yield, m.p 200–203 °C, [lit. [32], 203–2005 °C), [*α*]_observed_ + 0.65, [*α*]^20^_D_ + 130, (*c*, 0.5%, EtOH), lit. [32], [*α*]^20^_546_ + 176° (*c*, 0.70, EtOH)]. Hydrolysis of the mother liquor (1*S*,2*S*,1'*R*)-(+)-**3b** gave (1*S*,2*S*)-(−)-3-methylenecyclopropane-1,2-dicarboxylic acid {(1*S*,2*S*)-**3}** as an off-white solid in 90% yield, m.p. 201 203 °C, (lit. [32], m.p. 199.2–199.7 °C), [*α*]_observed_ + 1.25, [*α*]^20^_D_ − 125, (*c*, 0.5%, EtOH), lit. [32], [*α*]^25^_D_ − 131.3°; *c*, 0.81%, EtOH)], as shown in Scheme 3. The melting point, specific rotation values and the analytical data of compounds (1*R*,2*R*)-**3** and (1*S*,2*S*)-**3** were in agreement with those reported in the literature [32].

**Scheme 4:**
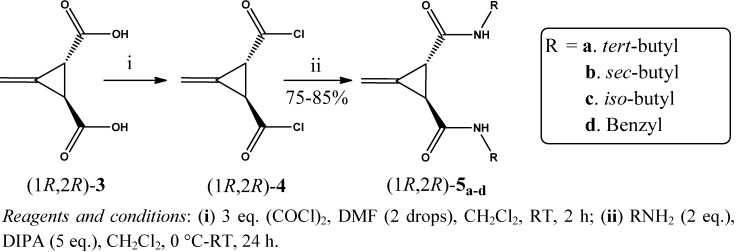
Synthesis of bis-amide ligands (1*R*,2*R*)**-5_a–d_** via acid chlorides.

**Scheme 5:**
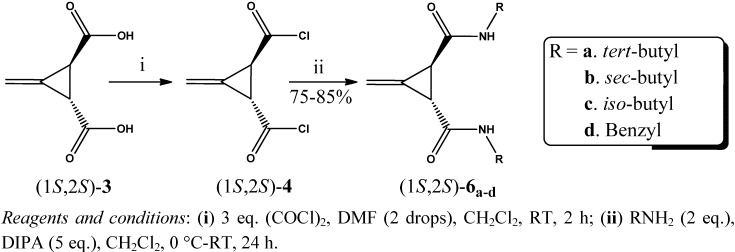
Synthesis of bis-amide ligands (1*S*,2*S*)**-6_a–d_** via acid chlorides.

*C_2_* Symmetric chiral bisamide ligands (1*R*,2*R*)-**5_a–d_** and (1*S*,2*S*)-**6_a–d_** have been synthesized in modest yield in two steps via the corresponding acid chloride intermediates, starting from the chiral Feist’s acids (1*R*,2*R*)-**3** and (1*S*,2*S*)-**3**, respectively, as described in Scheme 4 and Scheme 5. 

The syntheses of the acid chlorides (1*R*,2*R*)-**4** and (1*S*,2*S*)-**4** were carried out using two different procedures **A** and **B**, although the better yields of the ligands (up to 85%) were achieved by using the acid chloride obtained from procedure **B**, as described in the Experimental section. The formation of the acid chloride intermediates (1*R*,2*R*)-**4** and (1*S*,2*S*)-**4** was confirmed by IR spectroscopy, where the absence of a broad band around 3,452 cm^–1^ (due to OH stretching frequency) was observed, along with the shifting of the C=O stretching frequencies from 1,710 to 1,800 cm^–1^, indicating the formation of the acid chlorides. Furthermore, appearance of a sharp peak at 764 cm^–1^ was attributed to C–Cl stretching frequency.

The chiral bisamide ligands (1*R*,2*R*)-**5a** and (1*S*,2*S*)-**6a** were synthesized by treating two equivalents of *tert*-butyl amine with one equivalent of the freshly prepared acid chlorides (1*R*,2*R*)-**4** and (1*S*,2*S*)-**4**, respectively, in dry CH_2_Cl_2_ under inert conditions_,_ as shown in Scheme 4 and Scheme 5. The structures of both the compounds (1*R*,2*R*)-**5a** and (1*S*,2*S*)-**6a** were mainly deduced from their ^1^H-NMR spectra, where the presence of 18 protons at *δ* 1.24 and two protons at *δ* 7.87 as a singlet, corresponding to the 2×C(C**H**_3_)_3_ and 2×CON**H** groups, respectively, could be observed. These two ligands (1*R*,2*R*)-**5a** and (1*S*,2*S*)-**6a** were further confirmed by their IR spectra, where three characteristic sharp peaks were observed at 3,318 and 3,321, 1,641 and 1,642 and 1,551 and 1,549 cm^−1^ due to N–H(sec) stretching, C=O stretching and C–N stretching frequencies, respectively. The specific rotation values of the compounds (1*R*,2*R*)-**5a** and (1*S*,2*S*)-**6a** were found to be [*α*]^20^_D_ + 150° (*c*, 0.30%, 10% MeOH/CHCl_3_) and [*α*]^20^_D_ − 129 (*c*, 0.31%, 10% MeOH/CHCl_3_), respectively, in total agreement with their configuration.

The ligands (1*R*,2*R*)-**5b** and (1*S*,2*S*)-**6b** with a *sec*-butyl amide side chain, were obtained in good yield, in a similar fashion as described in the above paragraph (Scheme 4 and Scheme 5). Formation of these compounds were confirmed by IR and ^1^H-NMR spectroscopy. The IR spectra of these two ligands, showed sharp peaks at 3,273 and 3,269, 1,632 and 1,630 and 1,557 and 1,560 cm^–1^, which were assigned to the N–H (*sec*) stretching, C=O stretching and C–N stretching vibrations, respectively. In case of their ^1^H-NMR spectra, the presence of a triplet at *δ* 0.81, a doublet at *δ* 1.02 and another doublet at *δ* 8.05, which were assigned to the protons of two sets of primary methyls, secondary methyls and two sets of amide groups, respectively. The splitting of the NH proton is mainly due to the presence of an adjacent tertiary proton in the *sec*-butyl chain. The calculated specific rotation of the ligands (1*R*,2*R*)-**5b** and (1*S*,2*S*)-**6b** were found to be [*α*]^20^_D_ + 129 (*c*, 0.31%, 10% MeOH/CHCl_3_) and [*α*]^20^_D_ − 112 (*c*, 0.31%, 10% MeOH/CHCl_3_), respectively, which matching well with their configuration.

The ligands with the isobutyl chain (1*R*,2*R*)-**5c** and (1*S*,2*S*)-**6c** were obtained in very good yield, as described in the Experimental section (Scheme 4 and Scheme 5). The structures of these amide ligands were elucidated from their IR and ^1^H-NMR spectra. In the IR spectra, three sharp peaks were observed for both the ligands at 3,296 and 3,288, 1,632 and 1,634, and 1,558 and 1,559 cm^–1^, due to the stretching frequencies of *sec*-N–H, C=O and C–N groups, respectively. In the ^1^H-NMR, observation of a doublet at *δ* 0.84, and a triplet at *δ* 8.21, are indicative of the presence of four sets of primary methyls and two sets of amide groups, accordingly. The presence of two methylene protons in the *iso*-butyl chain adjacent to the NHCO, are responsible for the splitting of the NH proton signal into a triplet. The configuration of these ligands were determined from their specific rotation values, which were found to be [*α*]^20^_D_ + 76 (*c*, 0.26%, 10% MeOH/CHCl_3_) and [*α*]^20^_D_ − 133 (c, 0.30%, 10% MeOH/CHCl_3_), respectively.

Finally, the ligands (1*R*,2*R*)-**5d** and (1*S*,2*S*)-**6d** with benzyl groups in amide side chain were prepared in very satisfactory yield by reaction of benzylamine (2 eq.) with the intermediates (1*R*,2*R*)-**4** and (1*S*,2*S*)-**4**, respectively. These ligands were also characterized from the IR and ^1^H-NMR spectra. In the IR spectra of these two ligands, three characteristic sharp peaks at 3,289 and 3,297, 1,631 and 1,630 and 1,540 and 1,541 cm^–1^ were observed for the stretching frequencies of *sec*-N–H, C=O and C–N, respectively. The ^1^H-NMR of these ligands revealed characteristic peaks at *δ* 7.25–7.32 and 8.78 as a multiplet and triplet, which were assigned to the corresponding aromatic and amide protons, respectively. Again the splitting of the NH proton into a triplet was due to the presence of two benzylic protons in the amide chain. The configurations of these ligands were determined from their specific optical rotations. Thus the specific rotations for these two ligands were found to be [*α*]^20^_D_ + 100 (*c*, 0.4%, 10% MeOH/CHCl_3_) and [*α*]^20^_D_ − 166 (*c*, 0.31%, 10% MeOH/CHCl_3_), respectively.

In ^1^H-NMR spectra, for both types of ligands (1*R*,2*R*)-**5_a–d_** and (1*S*,2*S*)-**6_a–d_**, we found a shift of the NH proton’s *δ* values downfield to *δ* 7.87, 8.05, 8.21, and 8.78, and this downfield shift is due to the decreasing *+I* effect for the substituent groups in the amide chain in going from *tert*-butyl to a benzyl group.

## 3. Experimental

### 3.1. General

All the moisture and air sensitive reactions were carried out under an inert atmosphere using an argon filled glove box and standard Schlenk-line techniques. All the chemicals were purchased from Aldrich, Sigma-Aldrich and Fluka and were used as received without purification, unless otherwise stated. TLC plates were used for monitoring the reactions. Flash chromatography was carried out with silica gel (100–200 mesh). Pyridine, triethylamine and diisopropylamine were dried over sodium hydroxide. Diethyl ether and tetrahydrofuran were distilled from sodium benzophenone ketyl. Hexane, heptane and pentane were distilled by using sodium/triglyme benzophenone ketyl. Chloroform, dichloromethane, benzene, toluene and dimethyformamide were dried using calcium hydride. Deuterated solvents were dried over calcium hydride and deoxygenated prior to use. ^1^H and ^13^C-NMR spectra were recorded on Jeol-400 spectrometer (^1^H 400 MHz, ^13^C 100 MHz): using deuterated CHCl_3_ or DMSO as solvent. The chemical shifts (*δ* in ppm) for ^1^H- and ^13^C-NMR were referenced internally using residual non deuterated solvent resonance shift and reported to trimethylsilane (TMS). Coupling constants (*J*) are taken in Hertz (Hz). Specific rotations were measured by using ATAGO-POLAX-2L. Elemental analyses were performed on a Perkin Elmer 2400 Elemental Analyzer. IR spectra were recorded on a Model FTIR-800 Infrared FT-IR Spectrometer using KBr pellets for solids or neat for liquids. Mass spectrometric analysis was conducted by using ESI mode on AGILENT Technologies 6410-triple quad LC/MS instrument.

### 3.2. Resolution of trans-(±)-3-Methylenecyclopropane-1*,*2-dicarboxylic Acid *[(±)-**2**]*

*Synthesis of the Ammonium Salts of trans-(±)-3-Methylenecyclopropane-1*,*2-dicarboxylic Acids [(1R*,*2R*,*1'R)-(+)-***3a** **&**(*1S*,*2S*,*1'R*)-(−)-**3b**]. The *trans*-(*±*)-3-methylenecyclopropane-1,2-dicarboxylic acid [*±-*(2), 20 g, 0.14 mol) was added to an 8:2 mixture of *tert*-butanol-water (160 mL) and the suspension was heated to 90 °C on a steam bath until it had completely dissolved. The resulting solution was then removed from the steam bath and (*R*)-(+)-*α*-methylbenzamine (17 g, 0.14 mol) was added slowly over a period of 20 min. The reaction mixture was stirred for 10 min and left to stand for 24 h to give a mixture of (1*R*,2*R*,1'*R*)-(+)-**3a** and (1*S*,2*S*,1'*R*)-(+)-**3b** in 1:1 ratio.

*Isolation of the (R)-(+)-α-Methylbenzammonium Salt of (+)-3-Methylenecyclopropane-(1R*,*2R)-(+)-1*,*2-dicarboxylic Acid {(1R*,*2R*,*1'R)-(+)*-**3a**}. After keeping the solution of (1*R*,2*R*,1'*R*)-(+)-**3a** and (1*S*,2*S*,1'*R*)-(+)-**3b** at room temperature overnight, a white crystalline solid precipitated out, which was collected by filtration and washed with a cold mixture of *tert*-butyl alcohol and water (9:1, 2 × 80 mL). Finally the solid was dried to obtain the ammonium salt **3a**. Yield 18 g (68.1 mmol, 97.2 %, based on one of the diastereomeric Feist’s acid salts), m.p. 220–222 °C, [*α*]_observed_ + 0.55°, [*α*]^20^_D_ + 110° (*c*, 0.5%, H_2_O), (lit. [32], [*α*]^23.5^_D_ + 86.1°; *c*, 0.57%, H_2_O).

The crystalline material was recrystallized further from an 8:2 mixture of *tert*-butyl alcohol and water (60 mL) to afford fine crystals of the (*R*)-(+)-*α*-methylbenzammonium salt of (1*R*,2*R*)-(+)-3-methylenecyclopropane-1,2-dicarboxylic acid (1:1) {(1*R*,2*R*,1'*R*)-(+)-**3a**}; yield 17 g (64.32 mmol, 91.89%), based on the diastereomeric Feist’s acid salt), m.p. 219–222 °C, [*α*]_observed_ + 0.60°, [*α*]^20^_D_ + 120° (*c*, 0.50%, H_2_O), (lit. [32], [*α*]^25^_D_ + 94.7°; *c*, 0.61%, H_2_O). A small sample was further recrystallized, but no change of specific rotation and melting point was observed. Elemental analyses were in accordance with the reported literature [32]. 

*Resolution of the (*R*)-(+)-*α*-Methylbenzammonium Salt of (1S*,*2S)-(−)-3-Methylene-cyclopropane-1*,*2-dicarboxylic Acid (1:1) {(1S*,*2S*,*1'R)-(−)-***3b**} *and Regeneration of (*1S,2S*)-(−)-3-Methylenecyclopropane-1*,*2-dicarboxylic Acid* {(*1S*,*2S*)-**3**}. The mother liquor obtained after the isolation of solid (*R*)-(+)-*α*-methylbenzammonium salt of (1*R*,2*R*)-(+)-3-methylenecyclopropane-1,2-dicarboxylic acid (1:1){(1*R*,2*R*,1'*R*)-(+)-**3a**}, was concentrated under reduced pressure to give pale yellow coloured viscous solid (1*S*,2*S*,1'*R*)-(+)-**3b**(19.5 g, crude) which was dissolved in water (200 mL) and washed with ethyl acetate (2 × 100 mL) in order to remove the organic impurities. The aqueous phase was acidified with 1M H_2_SO_4_ and extracted with ethyl acetate (3 × 100 mL). The combined organic phase was then washed with brine (100 mL) and dried over anhydrous Mg_2_SO_4_. The organics were then concentrated under reduced pressure to afford (*1S*,*2S*)-(−)-3-methylenecyclopropane-1,2-dicarboxylic acid {(1*S*,2*S*)-**3**}, as an off white solid. Yield 9.0 g (63.38 mmol, 90%, based on one enantiomer). M.p. 201–203 °C, (lit. [32], m.p. 199.21–199.7 °C); [*α*]_observed_ + 1.25, [*α*]^20^_D_ − 125° (*c*, 0.5%, EtOH), (lit. [32], [*α*]25_D_ − 131.3°; *c*, 0.81%, EtOH); IR (cm^−1^): 3,500–2,500 (bs, OH str.), 1,700 (bs, C=O str.), 1,470 (s), 1,320 (s), 1,300 (s), 1,210 (s), 1,100 (s), 980 (s), 920 (s), 790 (s), 660 (m); ^1^H-NMR (DMSO-*d*_6_): *δ* 2.85 (s, 2H, C**H**), 5.9 (s, 2H, C=C**H**_2_), 12.9–13.2 (bs, 2H, COO**H**); ^13^C-NMR (DMSO-*d*_6_): *δ* 29.5 (**C**H), 110 (C=**C**H_2_), 133.5 (**C**=CH_2_), 174.0 (**C**OOH); Anal. Calcd. for C_6_H_6_O_4_: C, 50.71; H, 4.26. Found: C, 50.52; H, 4.33; LC/MS (EI^+^): *m/z* = 141.02 [M–H^+^].

*(1R*,*2R)-(+)-3-Methylenecyclopropane-1*,*2-dicarboxylic Acid {(1R*,*2R)-***3**: The (*R*)-(+)-*α*-methylbenzammonium salt of (1*R*,2*R*)-(+)-3-methylenecyclopropane-(+)-1,2-dicarb-oxylic acid (1:1) {(1*R*,2*R*,1'*R*)-(+)-**3a**}(17 g, 64.71 mmol) was dissolved in a 1:1 mixture of ethyl acetate and water (200 mL) and then acidified with 1 M H_2_SO_4_. The reaction mixture was stirred for 5 min then the organic phases were separated out. The aqueous phase was further extracted with 10% methanol in ethyl acetate (2 × 100 mL) and the combined organics were washed with brine (100 mL). Then the organics were dried over anhydrous magnesium sulfate and concentrated under reduced pressure to afford (1*R*,2*R*)-(+)-3-methylenecyclopropane-1,2-dicarboxylic acid {(1*R*,2*R*)-**3**}, as an off white solid. Yield 8.5 g (59.85 mmol, 92.5%, based on one enantiomer). 200–203 °C, (lit. [32], 203–205 °C), [*α*]_observed_ + 0.65°, [*α*]^20^_D_ + 130 (*c*, 0.5%, EtOH), (lit [32], [*α*]^20^_546_ + 176°; *c*, 0.70, EtOH); IR (cm^–1^): 3500–2500 (bs, OH str.), 1700 (bs, CO str.), 1470 (s), 1320 (s), 1300 (s), 1210 (s), 1100 (s), 980 (s), 920 (s), 790 (s), 660 (m); ^1^H-NMR (DMSO-*d*_6_): *δ* 2.85 (s, 2H, C**H**), 5.9 (s, 2H, C=C**H**_2_), 12.9–13.2 (bs, 2H, COO**H**); ^13^C-NMR (DMSO-*d*_6_): *δ* 29.5 (**C**H), 110 (C=**C**H_2_), 133.5 (**C**=CH_2_), 174.0 (**C**OOH); Anal. Calcd. for C_6_H_6_O_4_: C, 50.71; H, 4.26. Found: C, 50.52; H, 4.33; LC/MS (EI^+^): *m/z* = 141.02 [M−H^+^].

### 3.3. General Procedure for the Preparation of Acid Chlorides (1R*,*2R)-*4* and (1S,2S)*-**4** (**GP1**)*

**Procedure A**: In a 100 mL round bottom flask equipped with a condenser and inert atmosphere, Compound (1*R*,2*R*)-**3** or (1*S*,2*S*)-**3** (0.5 g, 3.52 mmol) was dissolved in benzene (20 mL) and 2 drops of DMF were added. Thionyl chloride (3 g, 14.08 mmol) was added to the reaction mixture at room temperature. The reaction mixture was heated to 60 °C for two hours, a clear wine red colour solution formed. The benzene was then removed under reduced pressure to afford crude acid chlorides (1*R*,2*R*)-**4** and (1*S*,2*S*)-**4** with quantitative yield ~100%. IR (cm^–1^): 1,800 cm^–1^ (C=O str.), absence of OH str. Frequency from 3,452 cm^–1^. 

**Procedure B**: In a 100 mL round bottom flask equipped with a condenser and inert atmosphere, Compound (1*R*,2*R*)-**3** or (1*S*,2*S*)-**3** (0.5 g, 3.52 mmol) was suspended in dry dichloromethane (20 mL) and 2 drops of DMF were added. Then oxallyl chloride (1.32 g, 10.56 mmol) was added dropwise to the reaction mixture at room temperature. The reaction mixture was stirred at ambient temperature for 1 h and it became the typical yellow colour of acid chloride solutions. The solvent was then removed under reduced pressure to afford crude acid chlorides (1*R*,2*R*)-**4** and (1*S*,2*S*)-**4** in very good yield (0.7 g, 3.53 mmol, ~100%, crude). IR (cm^–1^): 1,800 cm^–1^ (C=O str.), absence of OH str. vibration at 3,452 cm^–1^.

### 3.4. General Procedure for the Synthesis of Amides (1R*,*2R)*-**5_a–d_*** and (1S*,*2S)*-**6_a–d_** (**GP2**)*

A solution of compound (1*R*,2*R*)-**4** or (1*S*,2*S*)-**4** (0.7 g, 3.52 mmol) in CH_2_Cl_2_ (10 mL), was added slowly to the solution of amine **a**–**d** (2eq.) and diisopropyl amine (5 eq.) dissolved in CH_2_Cl_2_ (10 mL), at 0 to 5 °C. The reaction mixture was then allowed to stir at ambient temperature for 3 h. TLC showed (10% MeOH/CH_2_Cl_2_) complete consumption of starting material. The reaction mixture was then quenched with saturated solution of ammonium chloride (15 mL) and extracted with 10% methanol in chloroform (5 × 100 mL). The combine organic layers were washed with brine (100 mL) and dried over anhydrous magnesium sulfate. The solvent was removed under reduced pressure to afford crude material which was washed with diethyl ether to afford pure amides (1*R*,2*R*)-**5_a–d_** and (1*S*,2*S*)-**6_a–d_**.

*(1R*,*2R)-N1*,*N2-Tert-butyl-3-methylenecyclopropane-1*,*2-dicarboxamide* [(*1R*,*2R*)-**5a**]: Compound (1*R*,2*R*)-**5a** was obtained as a white solid by treating acid chloride (1*R*,2*R*)-**4** (3.52 mmol) with 2 eq. of *tert*-butylamine according to **GP2**. Yield 730 mg (2.9 mmol, 82.2%); m.p. 235–238 °C, [*α*]_observed_ + 0.45°, [*α*]^20^_D_ + 150°, (*c*, 0.30%, 10% MeOH/CHCl_3_); IR (cm^–1^): 3318 (s, sec N–H str.), 3073 (s), 2979 (s), 2961 (s), 1641 (s, C=O str.), 1551 (s, C–N str.), 1480 (s), 1360 (s), 1344 (s), 1249 (s), 1225 (s), 1108 (s), 890 (s), 652 (s); ^1^H-NMR (DMSO-*d*_6_): *δ* 1.24 (s, 18H, C(C**H**_3_)_3_), 2.68 (s, 2H, cyclopropyl C**H**), 5.37 (s, 2H, C=C**H**_2_), 7.87 (s, 2H, N**H**); ^13^C-NMR (DMSO-*d*_6_): *δ* 29.1 (**C**(CH_3_)_3_), 39.43 (C(**C**H_3_)_3_), 50.9 (cyclopropyl **C**H), 103.6 (C=**C**H_2_), 133.4 (**C**=CH_2_), 167.9 (NH**C**=O); Anal. Calcd. for C_14_H_24_N_2_O_2_ (252.18); C, 66.63; H, 9.59; N, 11.10; Found: C, 66.52; H, 9.83; N, 10.72; LC/MS (EI^+^): *m/z* = 253.1 [M+H^+^].

*(1R*,*2R)-N1*,*N2-sec-Butyl-3-methylenecyclopropane-1*,*2-dicarboxamide* [(*1R*,*2R*)-**5b**]: Compound (1*R*,2*R*)-**5b** was obtained as an off white solid by treating acid chloride (1*R*,2*R*)-**4** (3.52 mmol) with 2 eq. of *sec*-butylamine according to **GP2**. Yield 677 mg (2.68 mmol, 76%); m.p 235–238 °C, [*α*]_observed_ + 0.4, [*α*]^20^_D_ + 129, (*c*, 0.31%, 10% MeOH/CHCl_3_); IR (cm^–1^): 3273 (s, sec N–H str.), 3080 (s), 2968 (s), 2929 (s), 1632 (s, C=O str.), 1558 (s, C-N str.), 1451 (s), 1361 (s),1219 (s), 1107 (s), 903 (s), 723, (s), 670 (s); ^1^H-NMR (DMSO-*d*_6_): *δ* 0.82 (t, 6H, *J* = 7.32 Hz, CH_2_C**H**_3_), 1.02 (d, 6H, *J* = 6.6 Hz, CH(C**H**_3_)CH_2_), 1.37 (m, 4H, C**H**_2_CH_3_), 2.69 (s, 2H, cyclopropyl C**H**), 3.64 (m, 1H, C**H**(CH_3_)CH_2_), 5.40 (s, 2H, C=C**H**_2_), 8.06 (d, *J* = 7.72 Hz, 2H, N**H**); ^13^C-NMR (DMSO-*d*_6_): *δ* 11.0 (CH_2_**C**H_3_), 20.73 (CH**C**H_3_), 26.2 (**C**H_2_CH_3_), 29.5 (cyclopropyl **C**H), 46.6 (**C**HCH_3_), 103.9, (C=**C**H_2_), 133.16 (**C**=CH_2_), 167.8 (NH**C**=O); Anal. Calcd. for C_14_H_24_N_2_O_2_ (252.18); C, 66.63; H, 9.59; N, 11.10; Found: C, 65.99; H, 9.50; N, 10.11; LC/MS (EI^+^): *m/z* = 252.0 [M^+^].

*(1R*,*2R)-N1*,*N2-Isobutyl-3-methylenecyclopropane-1*,*2-dicarboxamide* [(*1R*,*2R*)-**5c**]: Compound (1*R*,2*R*)-**5c** was obtained as an off white solid by treating acid chloride (1*R*,2*R*)-**4** (3.52 mmol) with 2 eq. of *iso*-butylamine according to **GP2**. Yield 755 mg (3.00 mmol, 85%); m.p. 225–229 °C, [*α*]_observed_ + 0.2, [*α*]^20^_D_ + 76, (*c*, 0.26%, 10% MeOH/CHCl_3_); IR (cm^–1^): 3296 (s, sec N–H str.), 3084 (s), 2952 (s), 2913 (s), 1632 (s, C=O str.), 1558 (s, C-N str.), 1330 (s), 1212 (s), 1107 (s), 893 (s), 671 (s); ^1^H-NMR (DMSO-*d*_6_): *δ* 0.84 (d, 6H, *J* = 6.6 Hz, CH(C**H**_3_)_2_), 1.66 (m, 1H, C**H**(CH_3_)_2_), 2.74 (s, 2H, cyclopropyl C**H**), 2.89 (m, 2H, C**H**_2_CH(CH_3_)_2_), 5.41 (s, 2H, C=C**H**_2_), 8.21 (t, 2H, *J* = 5.36 Hz, N**H**); ^13^C-NMR (DMSO-*d*_6_): *δ* 20.1(CH(**C**H_3_)_2_), 25.6 (**C**H(CH_3_)_2_), 28.0 (cyclopropyl **C**H), 104.89 (C=**C**H_2_), 132.5 (**C**=CH_2_), 167.9 (NH**C**=O); Anal. Calcd. for C_14_H_24_N_2_O_2_ (252.18); C, 66.63; H, 9.59; N, 11.10; Found: C, 66.50; H, 9.77; N, 10.69; LC/MS (ES^+^): *m/z* = 253.2 [M+H^+^].

*(1R*,*2R)-N1*,*N2-Benzyl-3-methylenecyclopropane-1*,*2-dicarboxamide* [(*1R*,*2R*)-**5d**]: Compound (1*R*,2*R*)-**5d** was obtained as an off white solid by treating acid chloride (1*R*,2*R*)-**4**(3.52 mmol) with 2 eq. of benzylamine according to **GP2**. Yield 970 mg (3.1 mmol, 86%); 231–235 °C, [*α*]_observed_ + 0.4, [*α*]^20^_D_ + 100, (*c*, 0.4%, 10% MeOH/CHCl_3_); IR (cm^–1^): 3290 (s, sec-N–H str.), 1631 (s, C=O str.), 1540 (s, C–N str.), 1325 (s), 732 (s), 696 (s); ^1^H-NMR (DMSO-*d*_6_): *δ* 2.82 (s, 2H, cyclopropyl C**H**), 4.29 (d, 4H, *J* = 5.36 Hz, C**H**_2_Ph), 5.48 (s, 2H, C=C**H**_2_), 7.24–7.33 (m, 10H), 8.77 (t, 2H, *J* = 5.12 Hz, N**H**); ^13^C-NMR (DMSO-*d*_6_): *δ* 26.3 (cyclopropyl **C**H), 43.0 ((cyclopropyl **C**H), 104.6 (C=**C**H_2_), 127.4 (**C_4_** of aromatic), 127.9 (**C_2_** of aromatic), 128.9 (**C_3_** of aromatic), 132.6 (**C**=CH_2_), 139.7 (**C_1_** of aromatic ), 168.5 (NH**C=**O); Anal. Calcd. for C_20_H_20_N_2_O_2_ (320.15); C, 74.98; H, 6.29; N, 8.74; Found: C, 74.83; H, 6.47; N, 8.52; LC/MS (EI^+^): *m/z* = 321.15 [M+H^+^].

*(1S*,*2S)-N1*,*N2-tert-Butyl-3-methylenecyclopropane-1*,*2-dicarboxamide* [(*1S*,*2S*)-**6a**]: Compound (1*S*,2*S*)-**6a** was obtained as awhite solid by treating acid chloride (1*S*,2*S*)-**4** (3.52 mmol) with 2 eq. of *tert*-butylamine according to **GP2**. Yield 715 mg (2.83 mmol, 80.5%); m.p. 234–236 °C, [*α*]_observed_ − 0.40, [*α*]^20^_D_ − 129, (*c*, 0.31%, 10% MeOH/CHCl_3_); IR (cm^–1^): 3321 (s, *sec*-N–H str.), 3071 (s), 2979 (s), 2960 (s), 1642 (s, C=O str.), 1549 (s, C–N str.), 1481 (s), 1362 (s), 1344 (s), 1249 (s), 1228 (s), 1108 (s), 890 (s), 654 (s); ^1^H-NMR (DMSO-*d*_6_): *δ* 1.24 (s, 18H, C(C**H**_3_)_3_), 2.68 (s, 2H, cyclopropyl C**H**), 5.37 (s, 2H, C=C**H**_2_), 7.87 (s, 2H, N**H**); ^13^C-NMR (DMSO-*d*_6_): *δ* 29.09 (**C**(CH3)_3_), 39.4 (C(**C**H3)_3_), 50.9 (cyclopropyl **C**H), 103.6 (C=**C**H_2_), 133.38 (**C**=CH_2_), 167.9 (NH**C=**O); Anal. Calcd. for C_14_H_24_N_2_O_2_ (252.18); C, 66.63; H, 9.59; N, 11.10; Found: C, 66.62; H, 9.85; N, 10.91; LC/MS (EI^+^): *m/z* = 253.0 [M+H^+^].

*(1S*,*2S)-N1*,*N2-sec-butyl-3-methylenecyclopropane-1*,*2-dicarboxamide* [(*1S*,*2S*)-**6b**]: Compound (1*S*,2*S*)-**6b** was obtained as a white solid by treating acid chloride (1*S*,2*S*)-**4** (3.52 mmol) with 2 eq. of *sec*-butylamine according to **GP2**. Yield 690 mg (2.73 mmol, 77.73%); m.p. 234–236 °C, [*α*]_observed_ − 0.35, [*α*]^20^_D_ − 112, (*c*, 0.31%, 10% MeOH/CHCl_3_); IR (cm^–1^): 3269 (s, sec-N–H str.), 3081 (s), 2968 (s), 2930 (s), 1630 (s, C=O str.), 1560 (s, C–N str.), 1450 (s), 1361 (s),1219 (s), 1109 (s), 903 (s), 723, (s), 672 (s); ^1^H-NMR (DMSO-*d*_6_): *δ* 0.82 (t, 6H, *J* = 7.24 Hz, CH_2_C**H**_3_), 1.02 (d, 6H, *J* = 6.8 Hz, CH(C**H**_3_)CH_2_), 1.37 (m, 4H, C**H**_2_CH_3_), 2.69 (s, 2H, cyclopropyl C**H**), 3.64 (m, 1H, C**H**(CH_3_)CH_2_), 5.40 (s, 2H, C=C**H**_2_), 8.06 (d, 2H, *J* = 7.76 Hz, N**H**); ^13^C-NMR (DMSO-*d*_6_): *δ* 10.96 (CH_2_**C**H_3_), 20.73 (CH**C**H_3_), 26.18 (**C**H_2_CH_3_), 29.45 (cyclopropyl **C**H), 46.57 (**C**HCH_3_), 103.88 (C=**C**H_2_), 133.16 (**C**=CH_2_), 167.78 (NH**C=**O); Anal. Calcd. for C_14_H_24_N_2_O_2_ (252.18); C, 66.63; H, 9.59; N, 11.10; Found: C, 65.95; H, 9.51; N, 10.73; LC/MS (EI^+^): *m/z* = 252.2 [M^+^].

*(1S*,*2S)-N1*,*N2-isobutyl-3-methylenecyclopropane-1*,*2-dicarboxamide* [(*1S*,*2S*)-**6c**]: Compound (1*S*,2*S*)-**6c** was obtained as a white solid by treating acid chloride (1*S*,2*S*)-**4** (3.52 mmol) with 2 eq. of *iso*-butylamine according to **GP2**. Yield 740 mg (2.93 mmol, 83.36%); 226–230 °C, [*α*]_observed_ − 0.4, [*α*]^20^_D_ − 133, (*c*, 0.30%, 10% MeOH/CHCl_3_); IR (cm^–1^): 3288 (s, *sec*-N-H str.), 3085 (s), 2950 (s), 2917 (s), 1634 (s, C=O str.), 1559 (s, C-N str.), 1330 (s), 1211 (s), 1107 (s), 893 (s), 670 (s); ^1^H-NMR (DMSO-*d*_6_): *δ* 0.84 (d, 6H, *J* = 6.68 Hz, CH(C**H**_3_)_2_), 1.66 (m, 1H, C**H**(CH_3_)_2_), 2.74 (s, 2H, cyclopropyl C**H**), 2.89 (m, 2H, C**H**_2_CH(CH_3_)_2_), 5.41 (s, 2H, C=C**H**_2_), 8.21 (t, 2H, *J* = 5.24 Hz, N**H**); ^13^C-NMR (DMSO-*d*_6_): *δ* 20.1 (CH(**C**H_3_)_2_), 25.6 (**C**H(CH_3_)_2_), 28.0 (cyclopropyl **C**H), 104.9 (C=**C**H_2_), 132.47 (**C**=CH_2_), 167.9 (NH**C=**O); Anal. Calcd. for C_14_H_24_N_2_O_2_ (252.18); C, 66.63; H, 9.59; N, 11.10; Found: C, 66.55; H, 9.68; N, 10.93; LC/MS (EI^+^): *m/z* = 252.1 [M^+^].

*(1*S,2S*)-N^1^*,*N^2^-benzyl-3-methylenecyclopropane-1*,*2-dicarboxamide* [(*1S*,*2S*)-**6d**]: Compound (1*S*,2*S*)-**6d** was obtained as an off white solid by treating acid chloride (1*S*,2*S*)-**4** (3.52 mmol) with 2 eq. of benzylamine according to **GP2**. Yield 944 mg (2.94 mmol, 83.76%); m.p. 232–234 °C, [*α*]_observed_ − 0.5, [*α*]^20^_D_ − 166, (*c*, 0.31%, 10% MeOH/CHCl_3_); IR (cm^–1^): 3297 (s, sec-N–H str.), 1630 (s, C=O str.), 1541 (s, C–N str.), 1325 (s), 732 (s), 696 (s); ^1^H-NMR (DMSO-*d*_6_): *δ* 2.82 (s, 2H, cyclopropyl C**H**), 4.28 (d, 4H, *J* = 5.24 Hz, C**H**_2_Ph), 5.48 (s, 2H, C=C**H**_2_), 7.24–7.33 (m, 10H), 8.77 (t, 2H, *J* = 5.12 Hz, N**H**); ^13^C-NMR (DMSO-*d*_6_): *δ* 26.30 (cyclopropyl **C**H), 43.01 (cyclopropyl **C**H), 104.47 (C=**C**H_2_), 127.4 (**C_4_**, aromatic), 127.84 (**C_2_**, aromatic), 128.91 (**C_3_**, aromatic), 132.60 (**C**=CH_2_), 139.73 (**C_1_**, aromatic), 168.53 (NH**C=**O); Anal. Calcd. for C_20_H_20_N_2_O_2_ (320.15); C, 74.98; H, 6.29; N, 8.74; Found: C, 74.74; H, 6.43; N, 8.87; LC/MS (EI^+^): *m/z* = 321.15 [M+H^+^].

## 4. Conclusions

In conclusion, the synthesis of the new class of *C_2_* symmetric chiral bisamide ligands with high specific rotation values were carried out. All these ligands were derived from *trans*-(±)-3-methylene-cyclopropane-1,2-dicarboxylic acid (Feist’s acid), which served as the key precursor. In order to obtain the chiral backbone of the ligands, we have resolved *trans*-(±)-3-methylenecyclopropane-1,2-dicarboxylic acid in a new modified method, hence (1*R*,2*R*)-(+)-3-methylenecyclopropane-1,2-dicarboxylic acid and (1*S*,2*S*)-(−)-3-methylenecyclopropane-1,2-dicarboxylic acid were obtained with a high degree of enantioselectivity. The applications of these chiral ligands are under investigation and the results will be reported in the nearest future. On the basis of the chiral Feist’s acid, we are working to explore a large variety of novel classes of chiral *C_2_* symmetrical bidentate, or tetradentate ligands, with bulky environments.

## Supplementary Materials

Supplementary materials can be accessed at: http://www.mdpi.com/1420-3049/17/5/5550/s1
