# Supplementary material for: Synthesis and Characterization of Some New C2 Symmetric Chiral Bisamide Ligands Derived from Chiral Feist’s Acid
Source: Molecules. 2012 May 9;17(5):5550–63. doi: 10.3390/molecules17055550 (PMC6268764; doi:10.3390/molecules17055550)
Supplement: Supplementary file 1 [file molecules-17-05550-s001.pdf]

Molecules **2012**, *17*, 5550-5563; doi:10.3390/molecules17055550

OPEN ACCESS

molecules

ISSN 1420-3049

www.mdpi.com/journal/molecules

Article

Synthesis and Characterization of Some New C_2 Symmetric Chiral Bisamide Ligands Derived from Chiral Feist's Acid

Abdullah M. A. Al Majid *, Mohammad Shahidul Islam *, Zeid Abdullah Al-Othman, Ahlam F. Al-Salhoob and Assem Barakat

Department of Chemistry, Faculty of Science, King Saud University, P.O. Box 2455, Riyadh 11451, Saudi Arabia; E-Mails: zaothman@ksu.edu.sa (Z.A.A.-O.); rhofy29@hotmail.com (A.F.A.-S.); ambarakat@ksu.edu.sa (A.B.)

* Authors to whom correspondence should be addressed; E-Mails: amajid@ksu.edu.sa (A.M.A.A.M.); shahid.10amui@gmail.com (M.S.I.); Tel.: +9661-467-5889 (A.M.A.A.M.); Fax: +9661-467-5992 (A.M.A.A.M.); Tel.: +9665-467-5884 (M.S.I.); Fax: +9661-467-5992 (M.S.I.).

^{1}H-NMR and ^{13}C-NMR spectra of the prepared chiral compound $5_{a\text{-}d}$ and $6_{a\text{-}d}$ and Feist's acid.

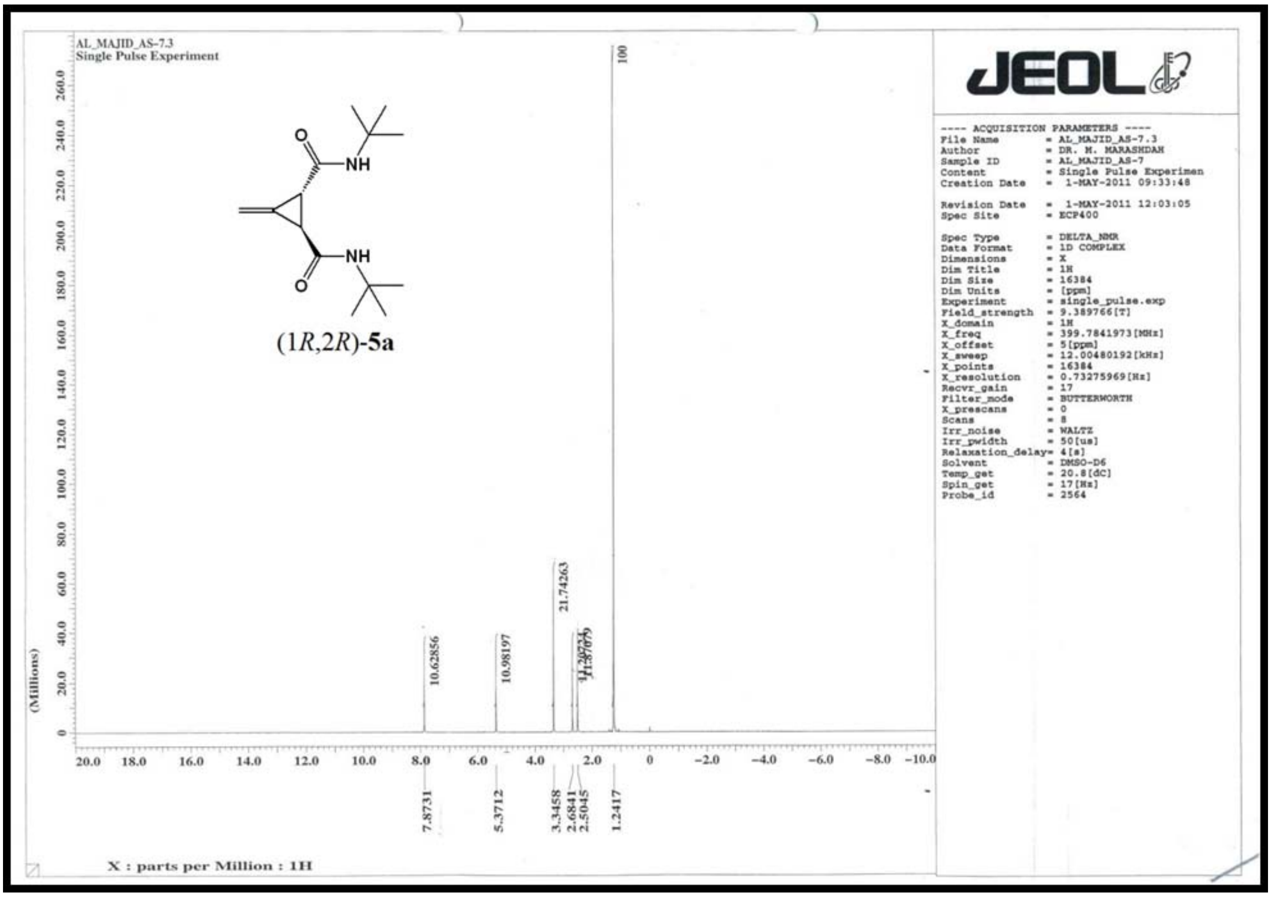

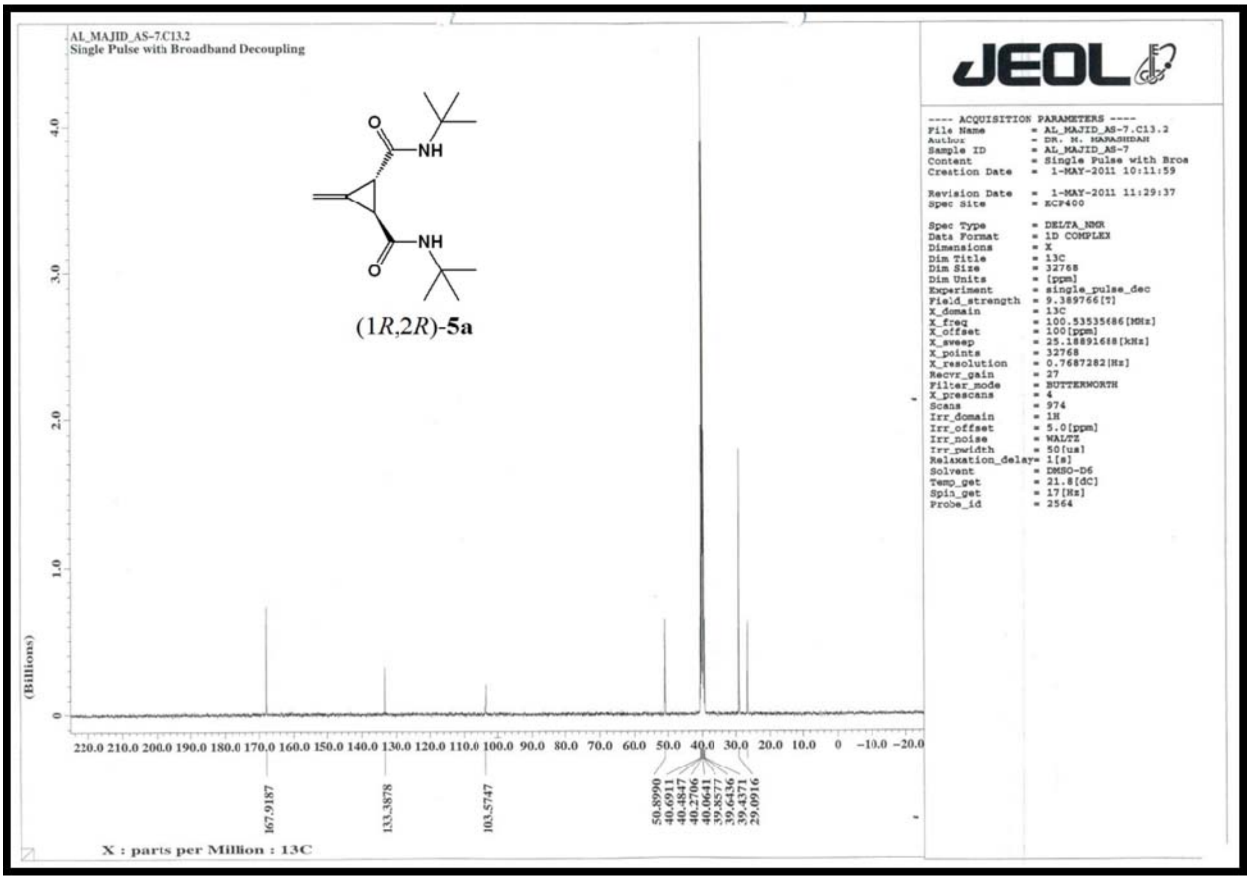
AL_MAJID_AS-7.C13.2
Single Pulse with Broadband Decoupling
(1*R*,2*R*)-**5a**
NH
NH
O
O
JEOL
---- ACQUISITION PARAMETERS ----
File Name = AL_MAJID_AS-7.C13.2
Author = DR. M. MARASHDAH
Sample ID = AL_MAJID_AS-7
Content = Single Pulse with Broa
Creation Date = 1-MAY-2011 10:11:59
Revision Date = 1-MAY-2011 11:29:37
Spec Site = ECP400
Spec Type = DELTA_NMR
Data Format = 1D COMPLEX
Dimensions = X
Dim Title = 13C
Dim Size = 32768
Dim Units = [ppm]
Experiment = single_pulse_dec
Field_strength = 9.389766[T]
X_domain = 13C
X_freq = 100.53535686[MHz]
X_offset = 100[ppm]
X_sweep = 25.18891688[kHz]
X_points = 32768
X_resolution = 0.7687282[Hz]
Recvr_gain = 27
Filter_mode = BUTTERWORTH
X_prescans = 4
Scans = 974
Irr_domain = 1H
Irr_offset = 5.0[ppm]
Irr_noise = WALTZ
Irr_pwidth = 50[us]
Relaxation_delay= 1[s]
Solvent = DMSO-D6
Temp_get = 21.8[dC]
Spin_get = 17[Hz]
Probe_id = 2564
(Billions)
167.9187
133.3878
103.5747
50.8990
40.6911
40.4847
40.2706
40.0641
39.8577
39.6436
39.4371
29.0916
X : parts per Million : 13C

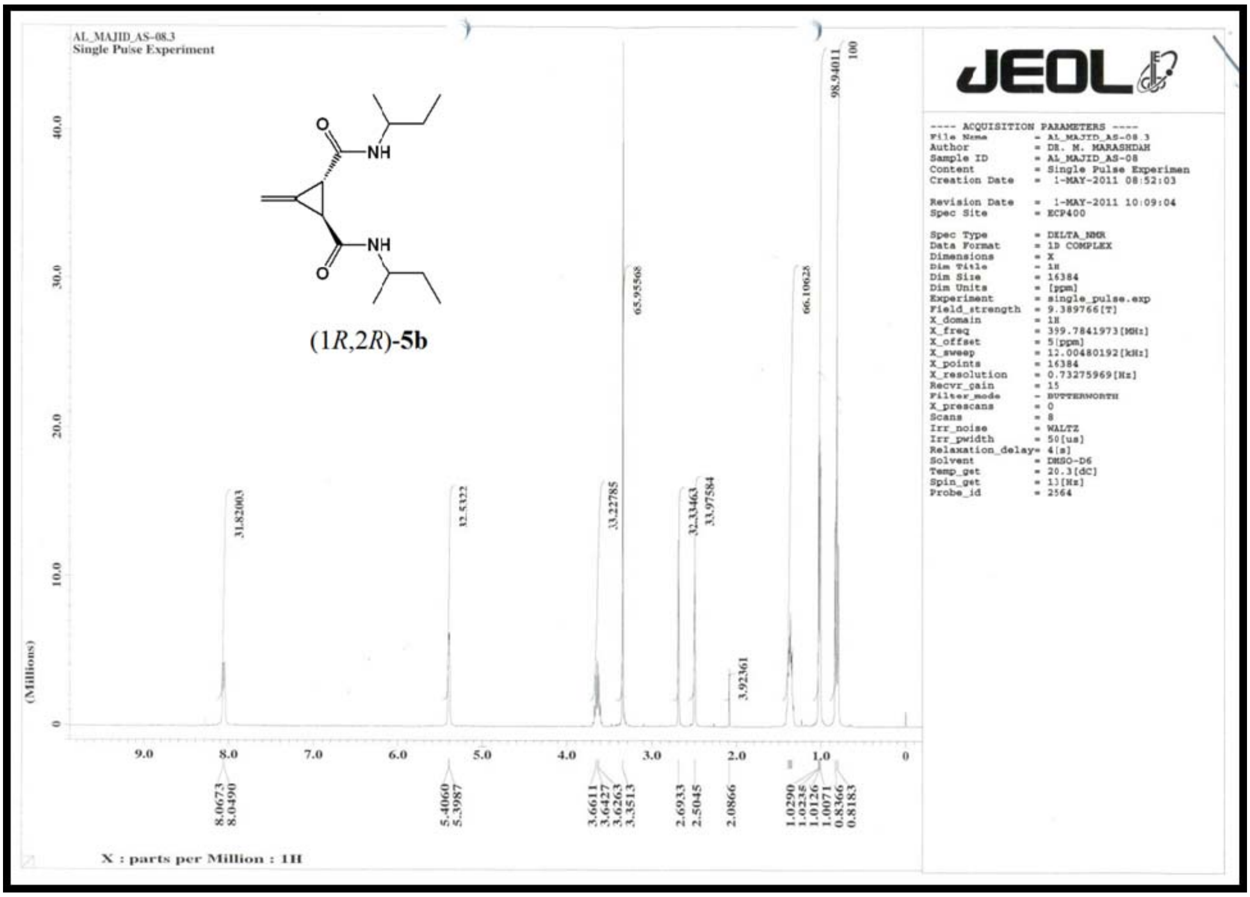
AL_MAJID_AS-08.3
Single Pulse Experiment
(1*R*,2*R*)-**5b**
NH
NH
O
O
JEOL
---- ACQUISITION PARAMETERS ----
File Name = AL_MAJID_AS-08.3
Author = DR. M. MARASHDAH
Sample ID = AL_MAJID_AS-08
Content = Single Pulse Experimen
Creation Date = 1-MAY-2011 08:52:03
Revision Date = 1-MAY-2011 10:09:04
Spec Site = ECP400
Spec Type = DELTA_NMR
Data Format = 1D COMPLEX
Dimensions = X
Dim Title = 1H
Dim Size = 16384
Dim Units = [ppm]
Experiment = single_pulse.exp
Field_strength = 9.389766[T]
X_domain = 1H
X_freq = 399.7841973[MHz]
X_offset = 5[ppm]
X_sweep = 12.00480192[kHz]
X_points = 16384
X_resolution = 0.73275969[Hz]
Recvr_gain = 15
Filter_mode = BUTTERWORTH
X_prescans = 0
Scans = 8
Irr_noise = WALTZ
Irr_pwidth = 50[us]
Relaxation_delay= 4[s]
Solvent = DMSO-D6
Temp_get = 20.3[dC]
Spin_get = 13[Hz]
Probe_id = 2564
(Millions)
31.82003
32.5322
33.22785
65.95568
32.33463
33.97584
3.92361
66.10628
98.94011
100
8.0673
8.0490
5.4060
5.3987
3.6611
3.6427
3.6263
3.3513
2.6933
2.5045
2.0866
1.0290
1.0238
1.0126
1.0071
0.8366
0.8183
X : parts per Million : 1H

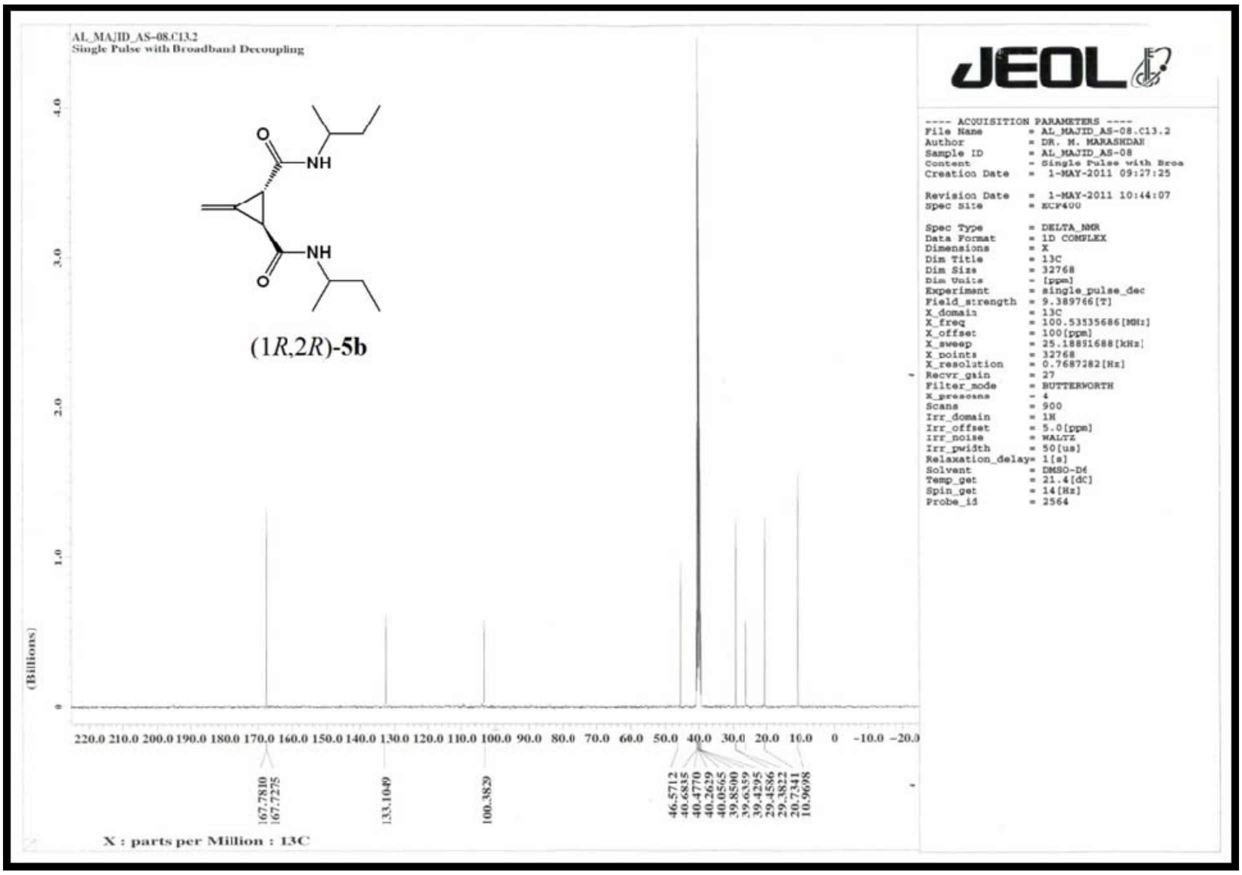
AL_MAJID_AS-08.C13.2
Single Pulse with Broadband Decoupling
(1R,2R)-5b
JEOL
X : parts per Million : 13C
167.7810
167.7275
133.1049
100.3829
46.5712
40.6835
40.4770
40.2629
40.0565
39.8500
39.6359
39.4295
29.4586
29.3822
20.7341
10.9698

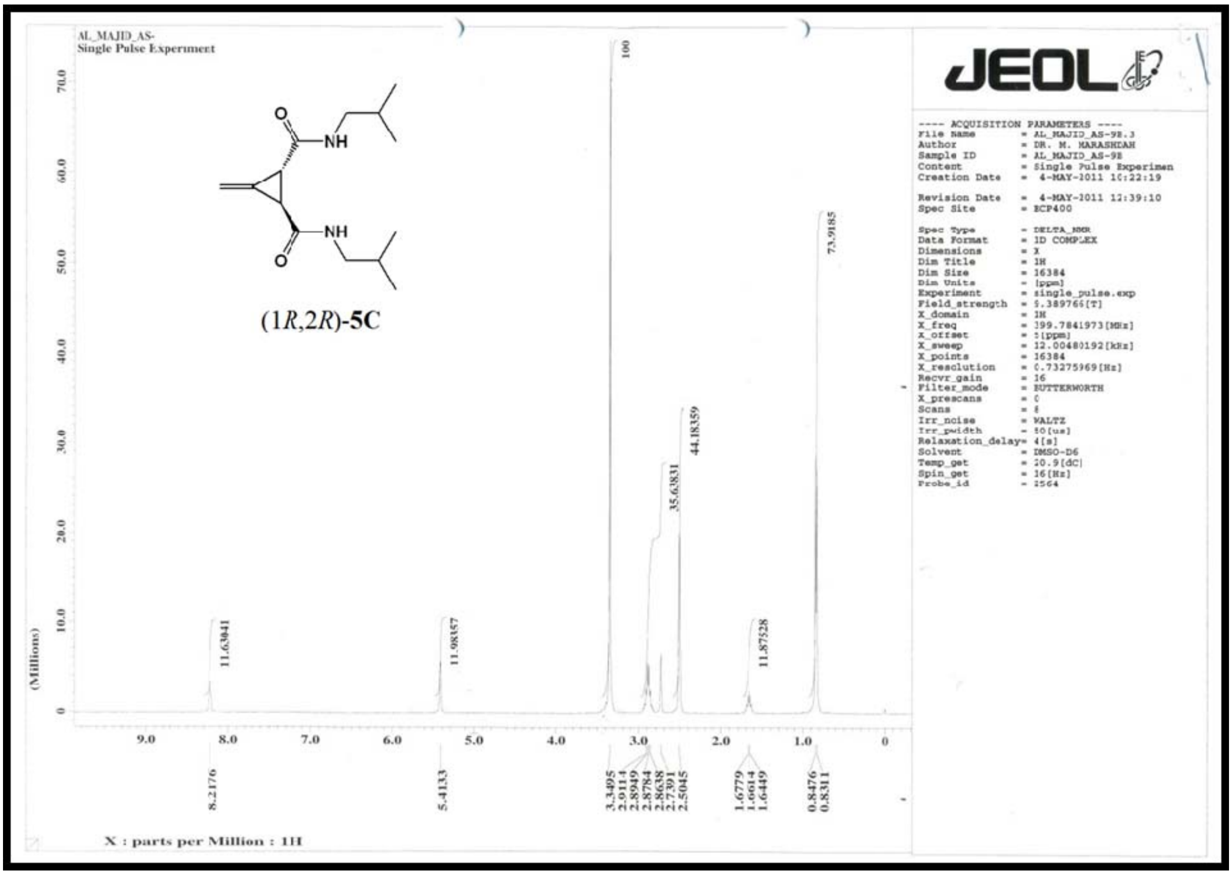
AL_MAJID_AS-
Single Pulse Experiment
(1R,2R)-5C
JEOL
X : parts per Million : 1H
8.2176
5.4133
3.3495
2.9114
2.8949
2.8784
2.8638
2.7391
2.5045
1.6779
1.6614
1.6449
0.8476
0.8311

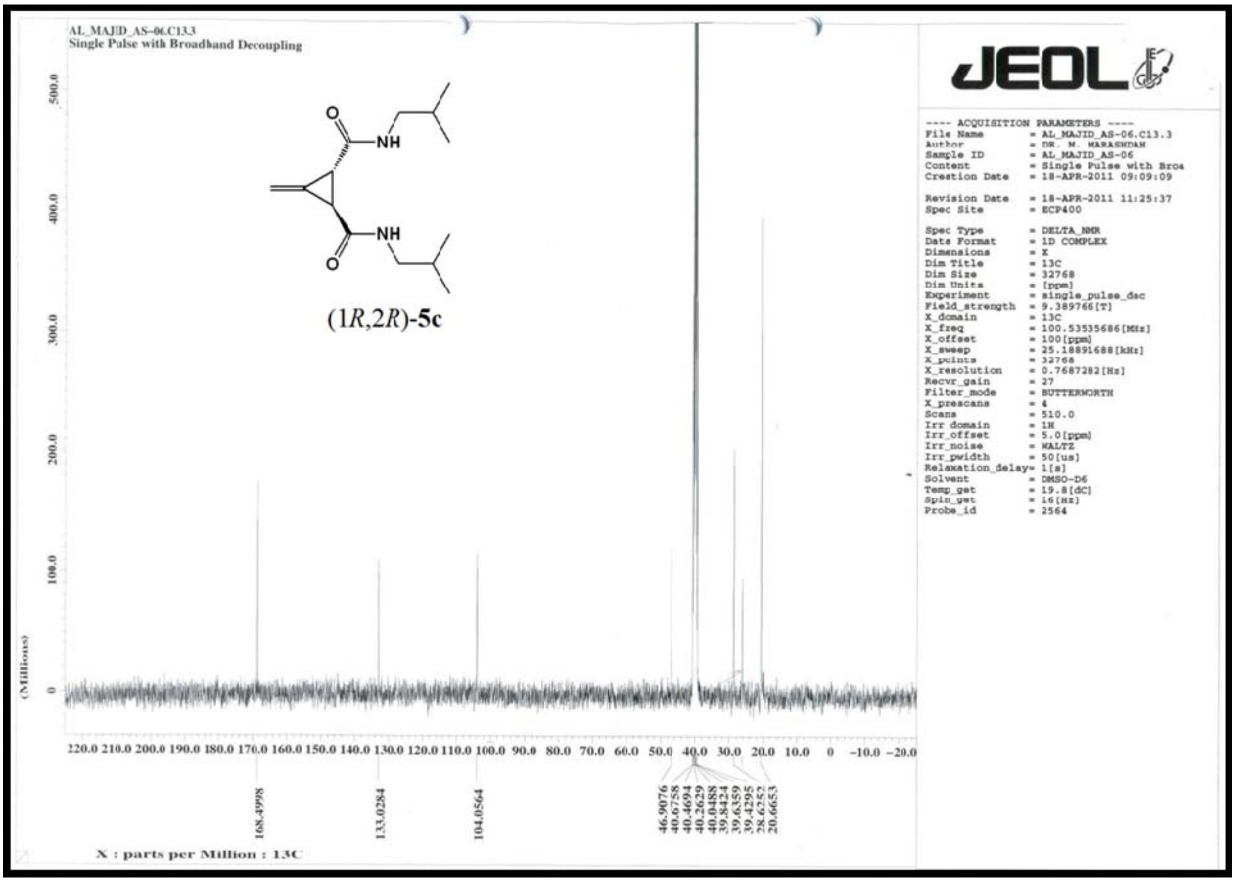

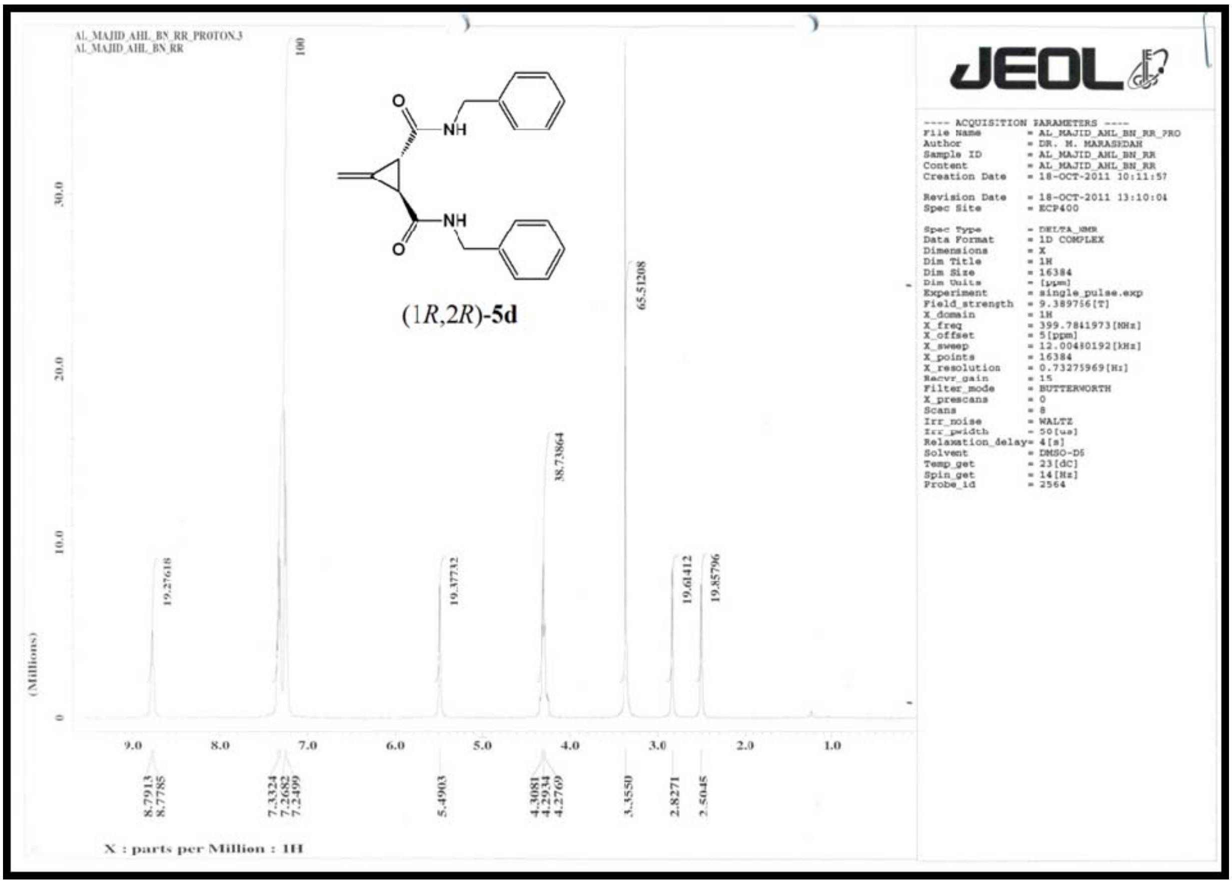

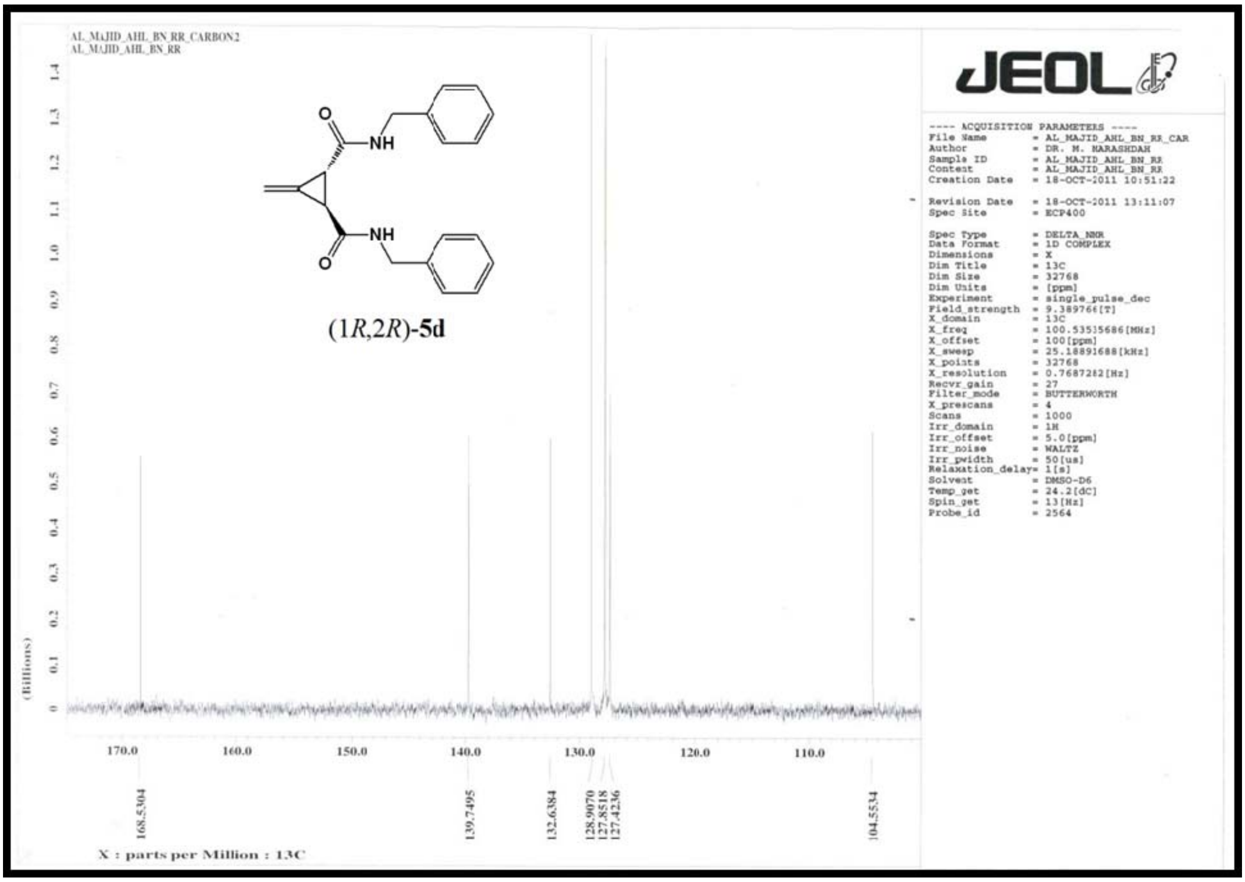
AL_MAJID_AHL_BN_RR_CARBON2
AL_MAJID_AHL_BN_RR
(1R,2R)-5d
JEOL
X : parts per Million : 13C
(Billions)
168.5304
139.7495
132.6384
128.9070
127.8518
127.4236
104.5534

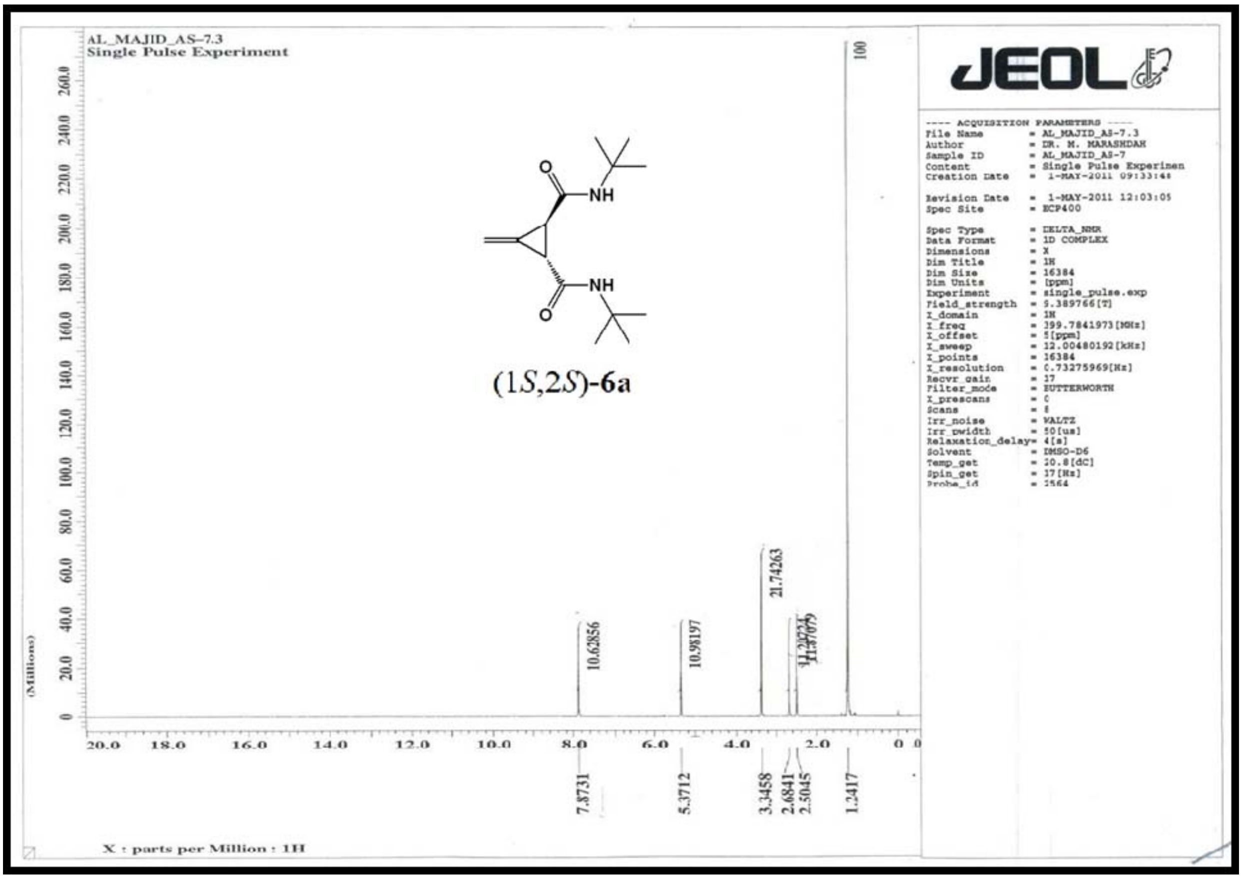
AL_MAJID_AS-7.3
Single Pulse Experiment
(1S,2S)-6a
JEOL
X : parts per Million : 1H
(Millions)
7.8731
5.3712
3.3458
2.6841
2.5045
1.2417

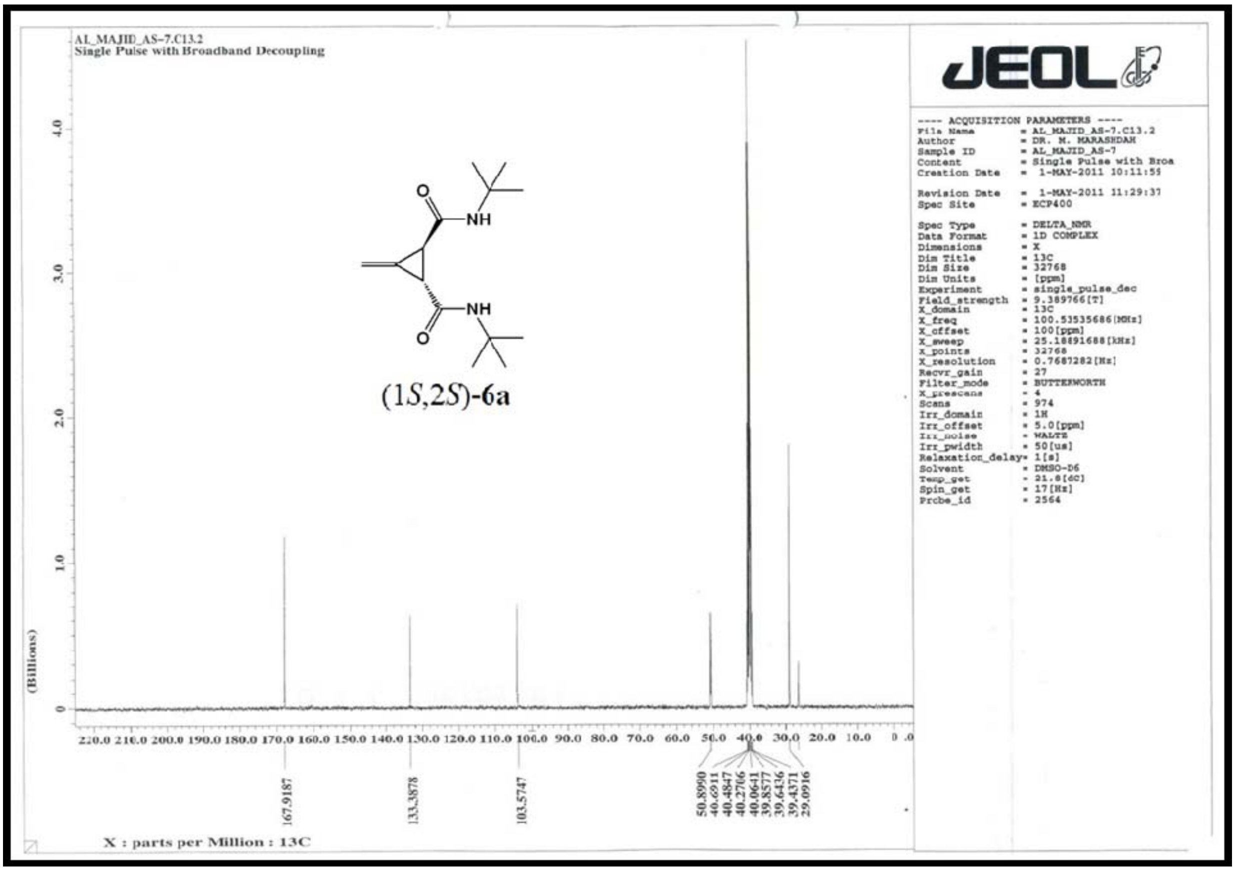
AL_MAJID_AS-7.C13.2
Single Pulse with Broadband Decoupling
JEOL
(1S,2S)-6a
X : parts per Million : 13C
167.9187
133.3878
103.5747
50.8990
40.6911
40.4847
40.2706
40.0641
39.8577
39.6436
39.4371
29.0916

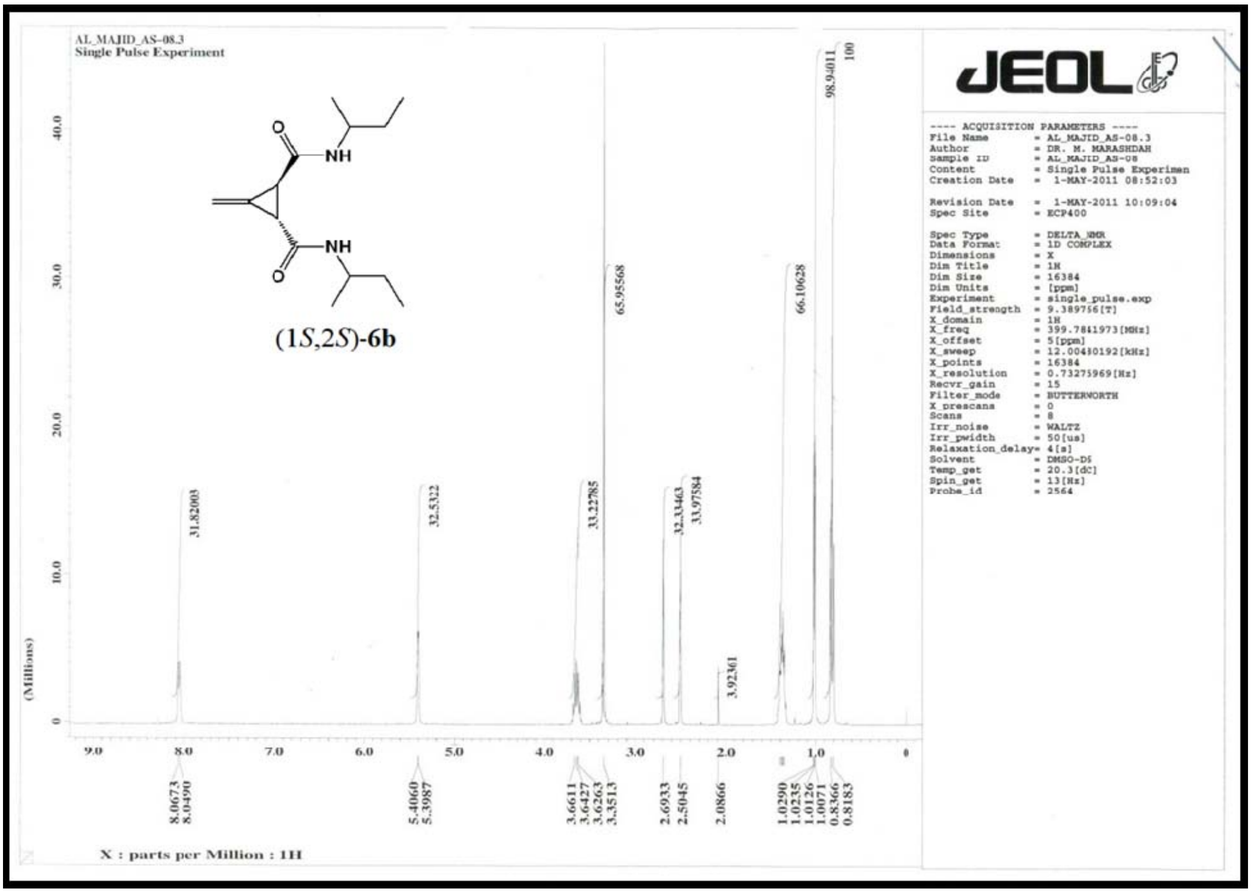
AL_MAJID_AS-08.3
Single Pulse Experiment
JEOL
(1S,2S)-6b
X : parts per Million : 1H
8.0673
8.0490
5.4060
5.3987
3.6611
3.6427
3.6263
3.3513
2.6933
2.5045
2.0866
1.0290
1.0235
1.0126
1.0071
0.8366
0.8183

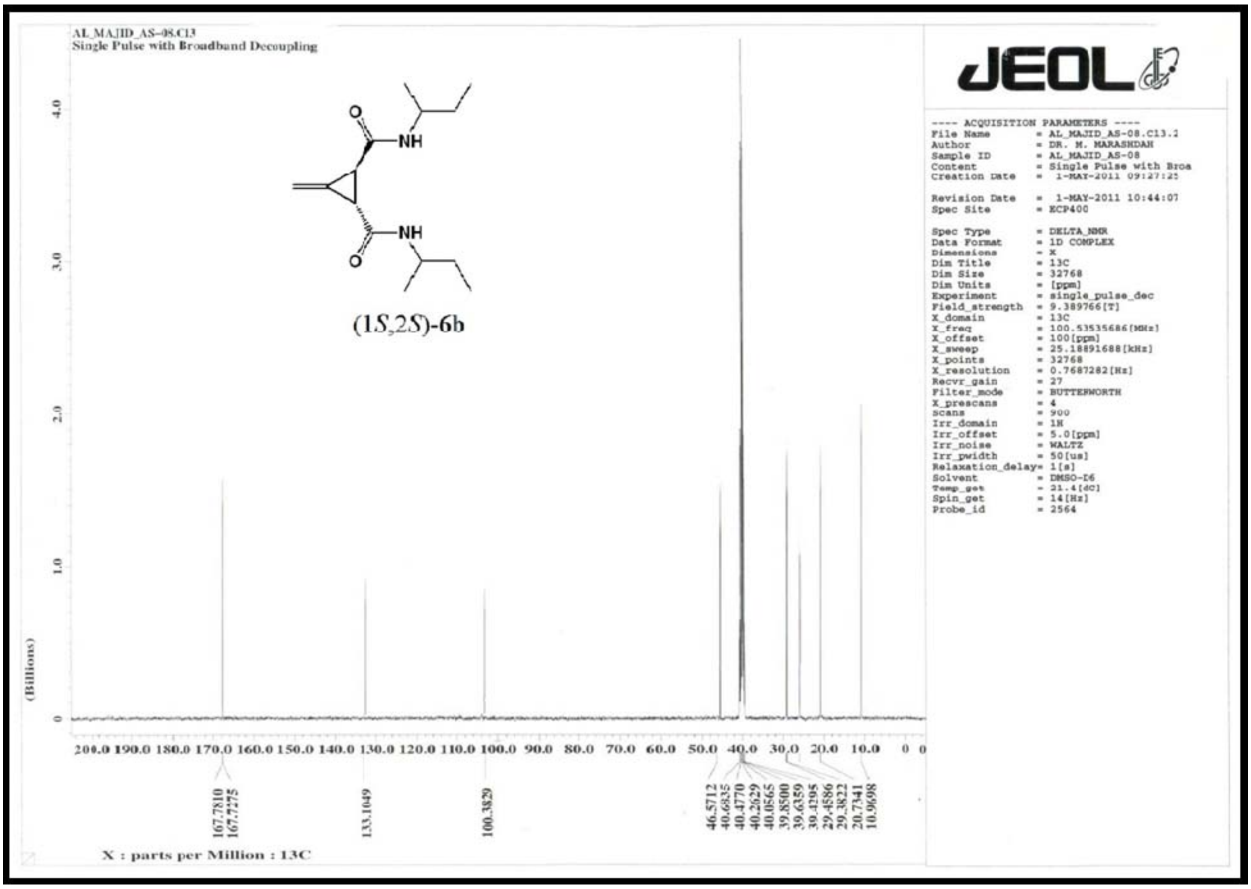
AL_MAJID_AS-08.C13
Single Pulse with Broadband Decoupling
O
NH
NH
O
(1S,2S)-6b
(Billions)
X : parts per Million : 13C
167.7810
167.7275
133.1049
100.3829
46.5712
40.6835
40.4770
40.2629
40.0565
39.8500
39.6359
39.4295
29.4586
29.3822
20.7341
10.9698
JEOL
---- ACQUISITION PARAMETERS ----
File Name = AL_MAJID_AS-08.C13.2
Author = DR. M. MARASHDAH
Sample ID = AL_MAJID_AS-08
Content = Single Pulse with Broa
Creation Date = 1-MAY-2011 09:27:25
Revision Date = 1-MAY-2011 10:44:07
Spec Site = ECP400
Spec Type = DELTA_NMR
Data Format = 1D COMPLEX
Dimensions = X
Dim Title = 13C
Dim Size = 32768
Dim Units = [ppm]
Experiment = single_pulse_dec
Field_strength = 9.389766[T]
X_domain = 13C
X_freq = 100.53535686[MHz]
X_offset = 100[ppm]
X_sweep = 25.18891688[kHz]
X_points = 32768
X_resolution = 0.7687282[Hz]
Recvr_gain = 27
Filter_mode = BUTTERWORTH
X_prescans = 4
Scans = 900
Irr_domain = 1H
Irr_offset = 5.0[ppm]
Irr_noise = WALTZ
Irr_pwidth = 50[us]
Relaxation_delay = 1[s]
Solvent = DMSO-D6
Temp_get = 21.4[dC]
Spin_get = 14[Hz]
Probe_id = 2564

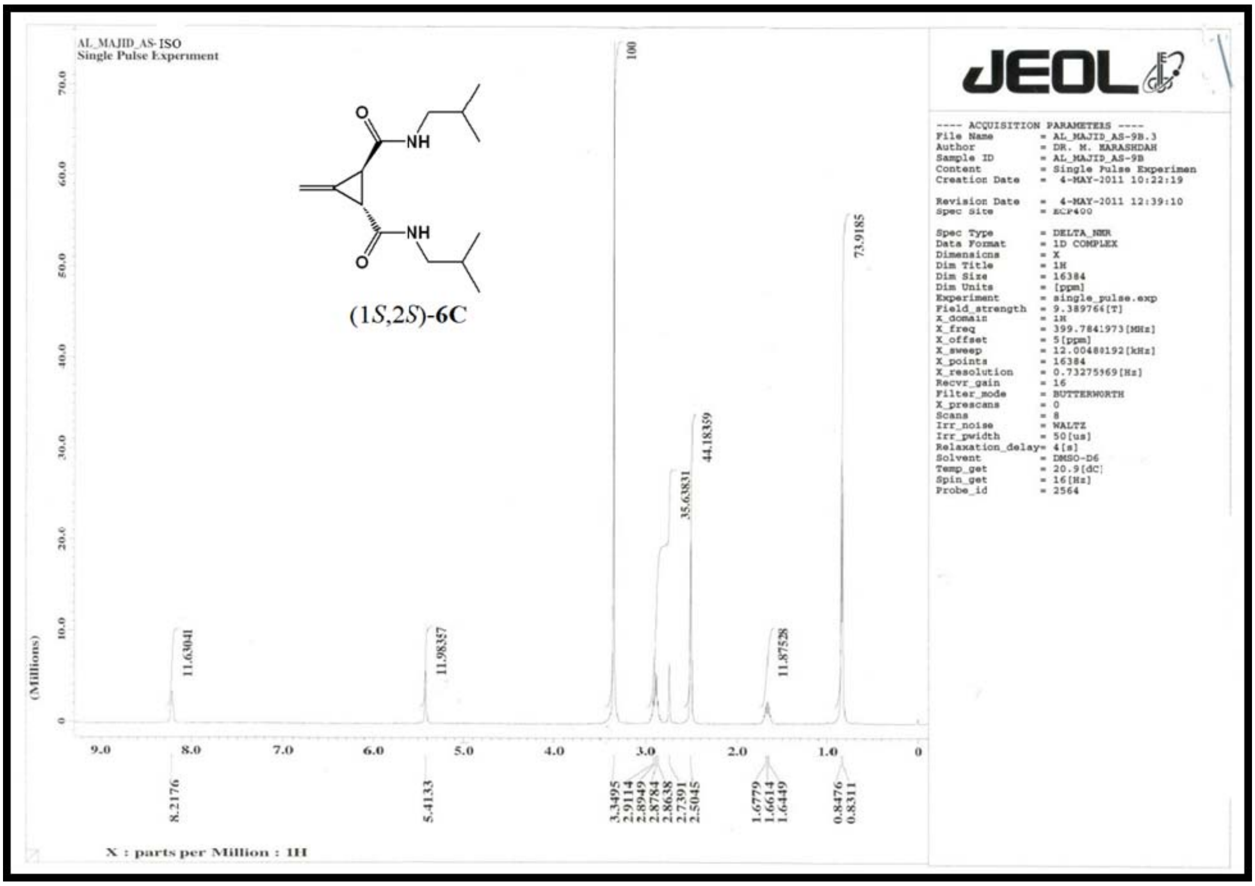
AL_MAJID_AS-ISO
Single Pulse Experiment
O
NH
NH
O
(1S,2S)-6C
(Millions)
X : parts per Million : 1H
100
73.9185
44.18359
35.63831
11.63041
11.98357
11.87528
8.2176
5.4133
3.3495
2.9114
2.8949
2.8784
2.8638
2.7391
2.5045
1.6779
1.6614
1.6449
0.8476
0.8311
JEOL
---- ACQUISITION PARAMETERS ----
File Name = AL_MAJID_AS-9B.3
Author = DR. M. MARASHDAH
Sample ID = AL_MAJID_AS-9B
Content = Single Pulse Experimen
Creation Date = 4-MAY-2011 10:22:19
Revision Date = 4-MAY-2011 12:39:10
Spec Site = ECP400
Spec Type = DELTA_NMR
Data Format = 1D COMPLEX
Dimensions = X
Dim Title = 1H
Dim Size = 16384
Dim Units = [ppm]
Experiment = single_pulse.exp
Field_strength = 9.389766[T]
X_domain = 1H
X_freq = 399.7841973[MHz]
X_offset = 5[ppm]
X_sweep = 12.00480192[kHz]
X_points = 16384
X_resolution = 0.73275969[Hz]
Recvr_gain = 16
Filter_mode = BUTTERWORTH
X_prescans = 0
Scans = 8
Irr_noise = WALTZ
Irr_pwidth = 50[us]
Relaxation_delay = 4[s]
Solvent = DMSO-D6
Temp_get = 20.9[dC]
Spin_get = 16[Hz]
Probe_id = 2564

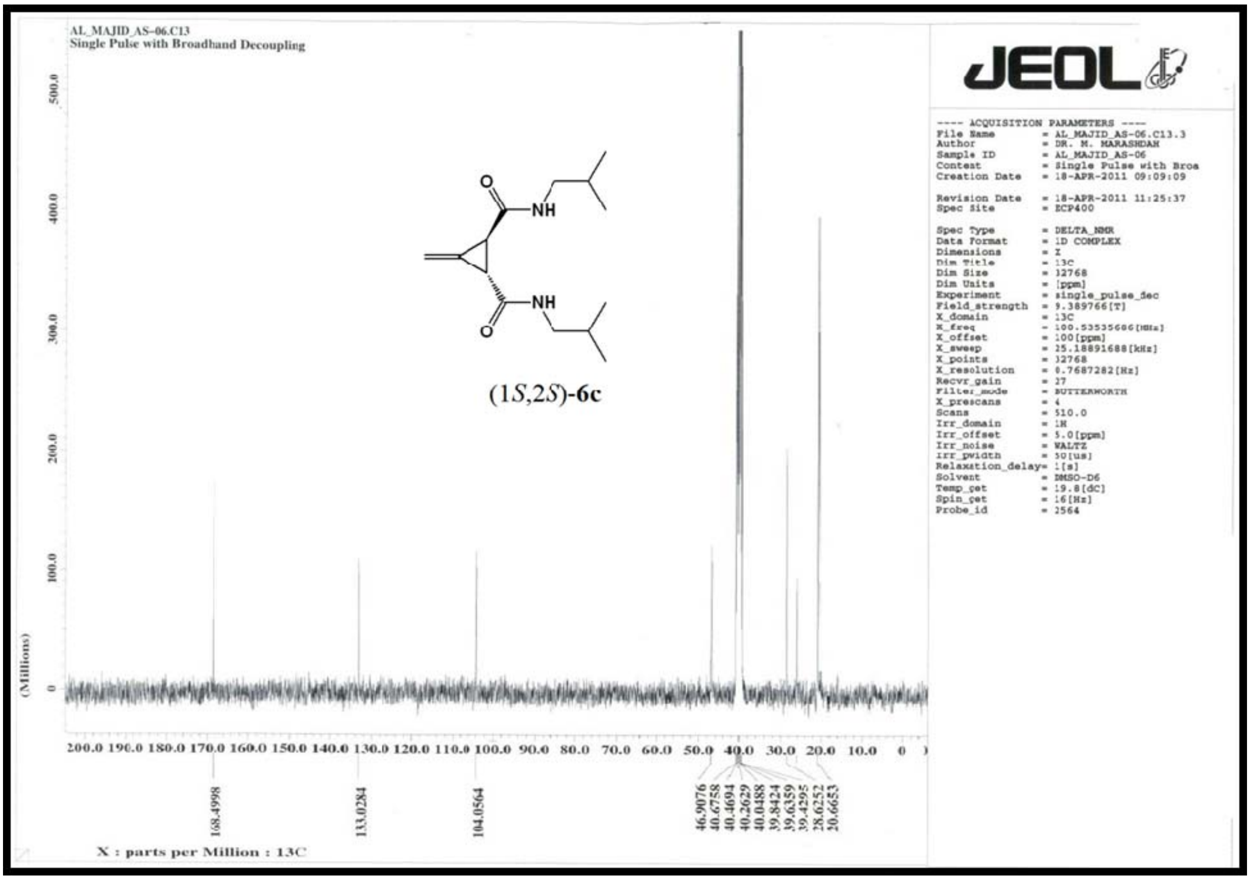
AL_MAJID_AS-06.C13
Single Pulse with Broadband Decoupling
(1S,2S)-6c
JEOL
X : parts per Million : 13C
168.4998
133.0284
104.0564
46.9076
40.6758
40.4694
40.2629
40.0488
39.8424
39.6359
39.4295
28.6252
20.6653
---- ACQUISITION PARAMETERS ----
File Name = AL_MAJID_AS-06.C13.3
Author = DR. M. MARASHDAH
Sample ID = AL_MAJID_AS-06
Content = Single Pulse with Broa
Creation Date = 18-APR-2011 09:09:09
Revision Date = 18-APR-2011 11:25:37
Spec Site = ECP400
Solvent = DMSO-D6

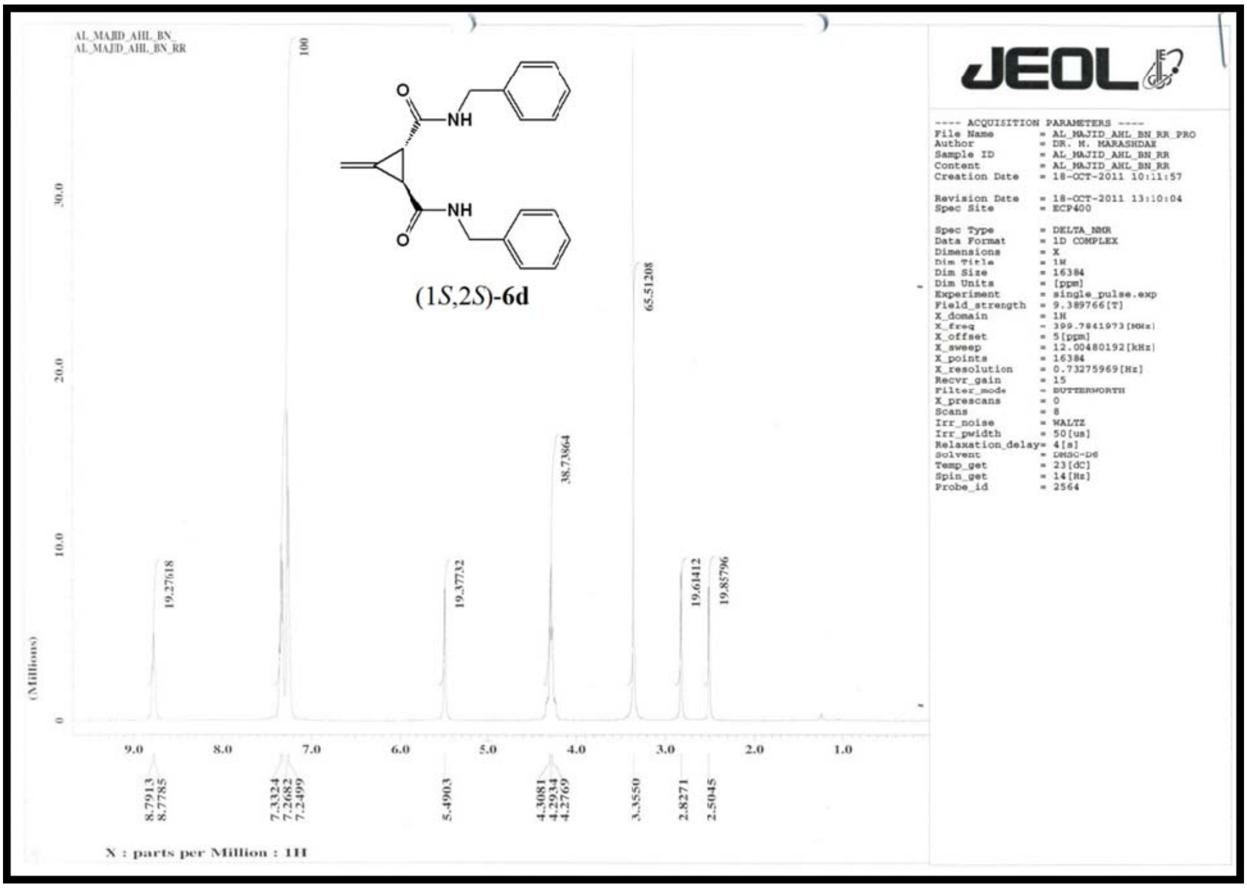
AL_MAJID_AHL_BN_
AL_MAJID_AHL_BN_RR
(1S,2S)-6d
JEOL
X : parts per Million : 1H
8.7913
8.7785
7.3324
7.2682
7.2499
5.4903
4.3081
4.2934
4.2769
3.3550
2.8271
2.5045
---- ACQUISITION PARAMETERS ----
File Name = AL_MAJID_AHL_BN_RR_PRO
Author = DR. M. MARASHDAH
Sample ID = AL_MAJID_AHL_BN_RR
Content = AL_MAJID_AHL_BN_RR
Creation Date = 18-OCT-2011 10:11:57
Revision Date = 18-OCT-2011 13:10:04
Spec Site = ECP400
Solvent = DMSO-D6

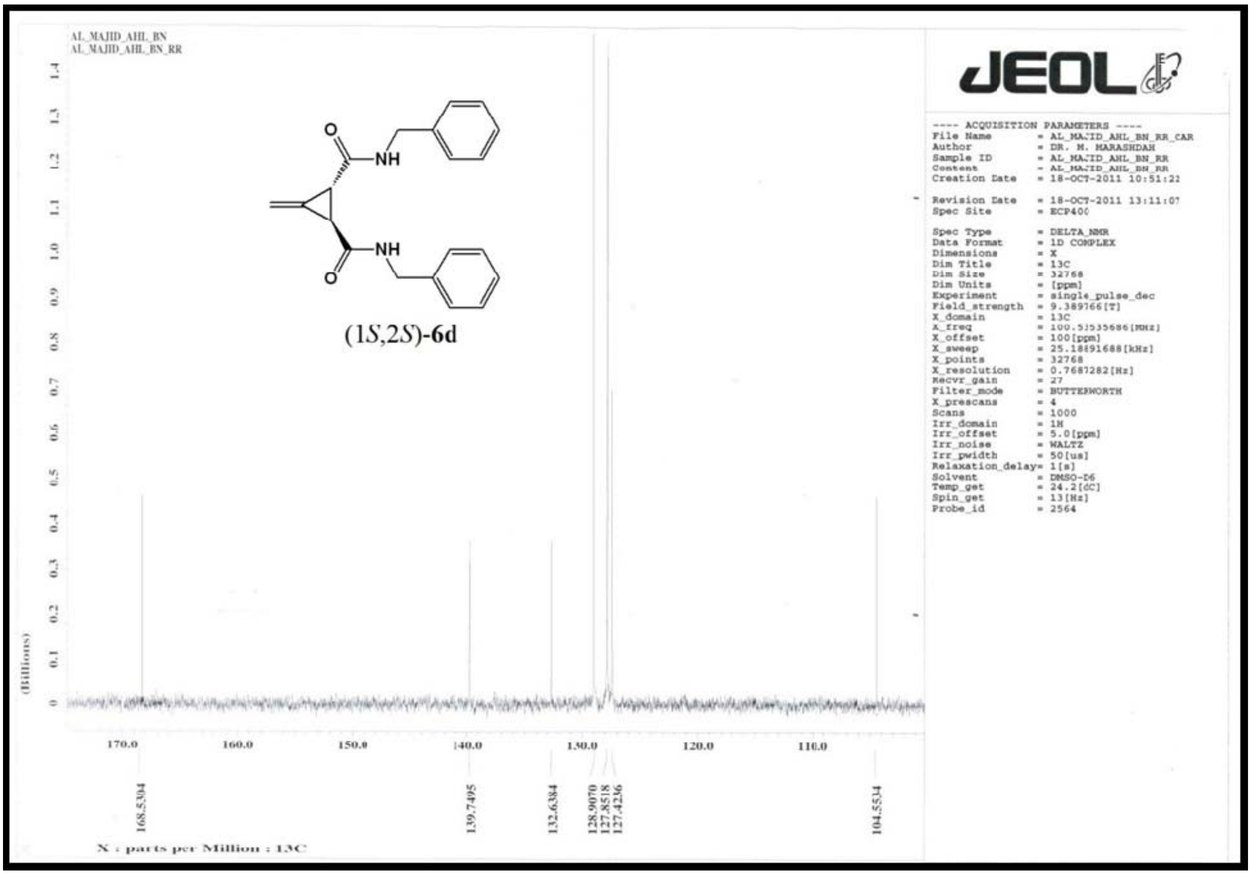
AL_MAJID_AHL_BN
AL_MAJID_AHL_BN_RR
O
NH
NH
O
(1*S*,2*S*)-**6d**
JEOL
---- ACQUISITION PARAMETERS ----
File Name = AL_MAJID_AHL_BN_RR_CAR
Author = DR. M. MARASHDAH
Sample ID = AL_MAJID_AHL_BN_RR
Content = AL_MAJID_AHL_BN_RR
Creation Date = 18-OCT-2011 10:51:22
Revision Date = 18-OCT-2011 13:11:07
Spec Site = ECP400
Spec Type = DELTA_NMR
Data Format = 1D COMPLEX
Dimensions = X
Dim Title = 13C
Dim Size = 32768
Dim Units = [ppm]
Experiment = single_pulse_dec
Field_strength = 9.389766[T]
X_domain = 13C
X_freq = 100.52535686[MHz]
X_offset = 100[ppm]
X_sweep = 25.18891688[kHz]
X_points = 32768
X_resolution = 0.7687282[Hz]
Recvr_gain = 27
Filter_mode = BUTTERWORTH
X_prescans = 4
Scans = 1000
Irr_domain = 1H
Irr_offset = 5.0[ppm]
Irr_noise = WALTZ
Irr_pwidth = 50[us]
Relaxation_delay= 1[s]
Solvent = DMSO-D6
Temp_get = 24.2[dC]
Spin_get = 13[Hz]
Probe_id = 2564
(Billions)
170.0
160.0
150.0
140.0
130.0
120.0
110.0
168.5304
139.7495
132.6384
128.9070
127.8518
127.4236
104.5534
X : parts per Million : 13C

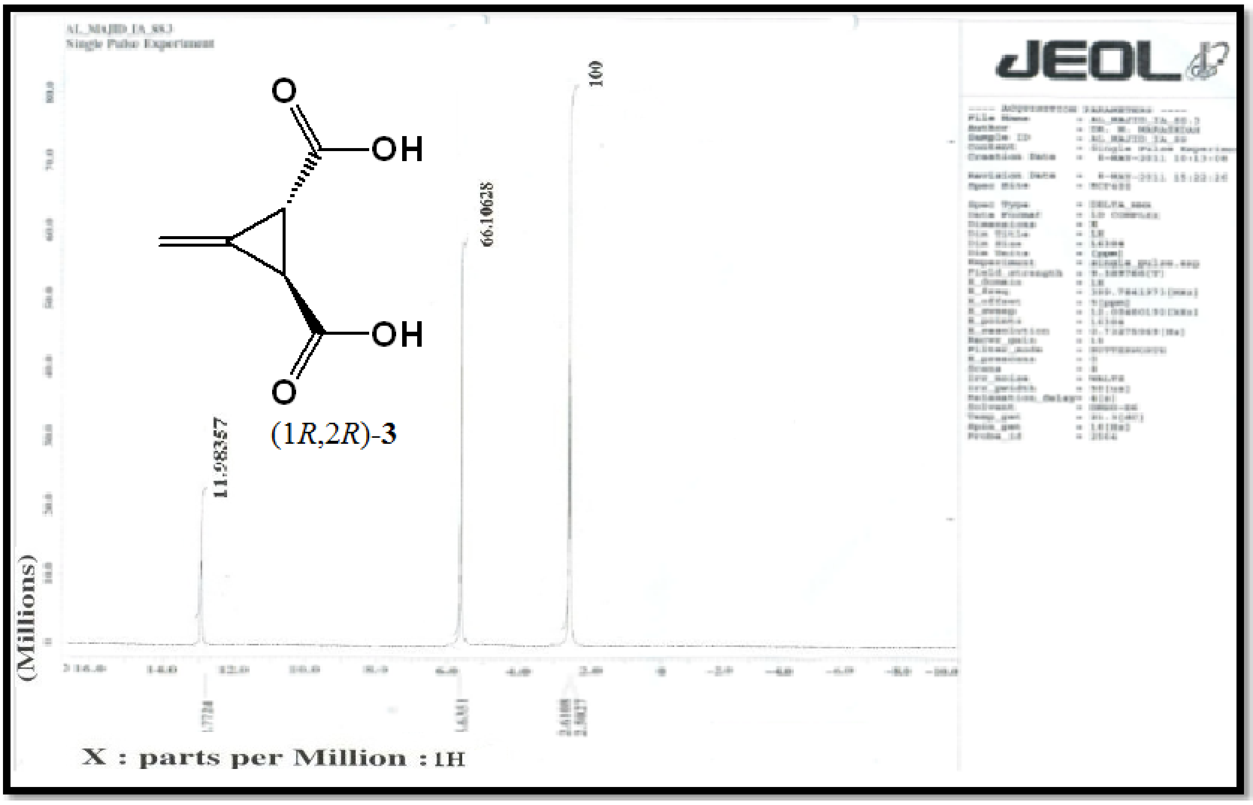
O
OH
OH
O
(1*R*,2*R*)-**3**
JEOL
100
66.10628
11.98357
(Millions)
X : parts per Million : 1H

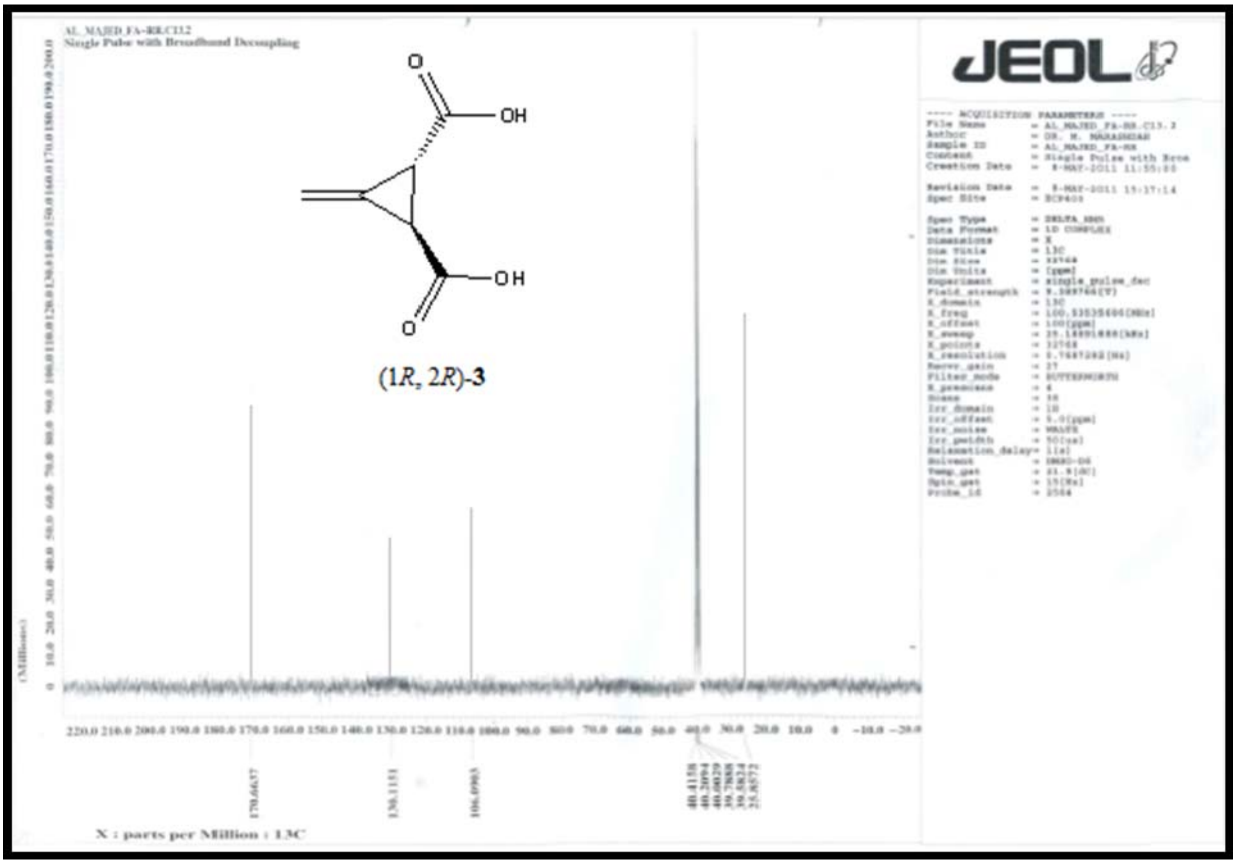
Single Pulse with Broadband Decoupling
OH
OH
(1R, 2R)-3
JEOL
170.6637
130.1151
106.0903
X : parts per Million : 13C

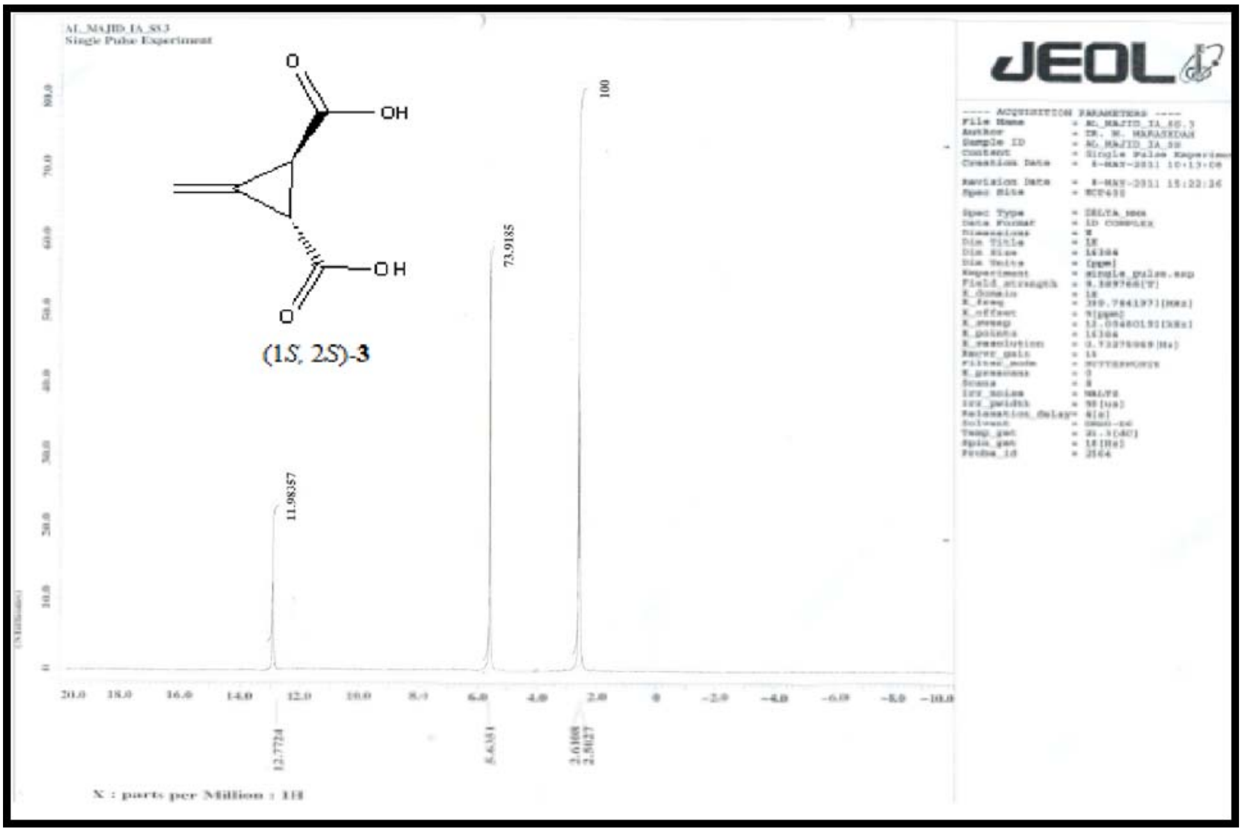
Single Pulse Experiment
OH
OH
(1S, 2S)-3
JEOL
11.98357
73.9185
100
12.7724
X : parts per Million : 1H

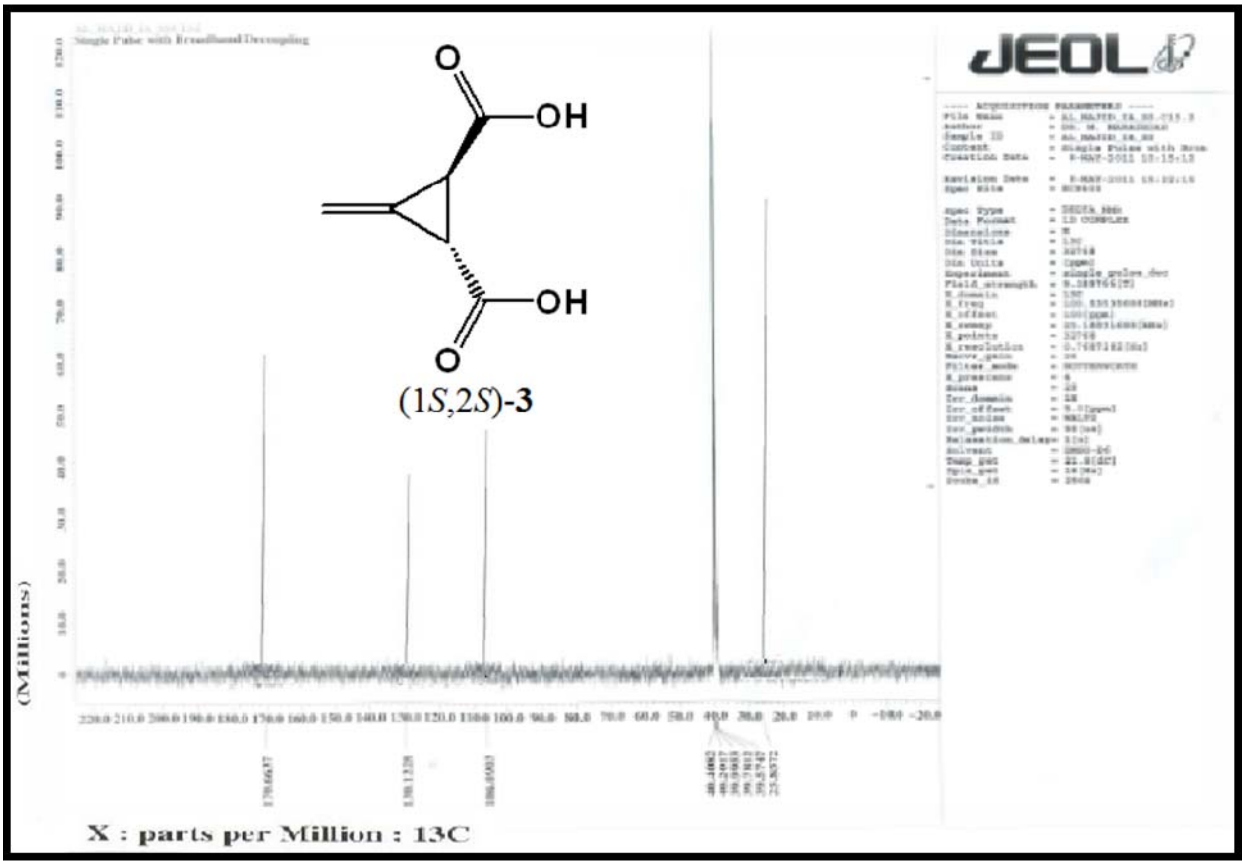
Single Pulse with Broadband Decoupling
O
OH
OH
O
(1S,2S)-3
JEOL
(Millions)
X : parts per Million : 13C
170.6637
130.1328
106.0503
40.1602
39.9583
25.8572
